# Deep learning-aided decision support for diagnosis of skin disease across skin tones

**DOI:** 10.1038/s41591-023-02728-3

**Published:** 2024-02-05

**Authors:** Matthew Groh, Omar Badri, Roxana Daneshjou, Arash Koochek, Caleb Harris, Luis R. Soenksen, P. Murali Doraiswamy, Rosalind Picard

**Affiliations:** 1 Northwestern University Kellogg School of Management, Evanston, IL USA; 2 MIT Media Lab, Cambridge, MA USA; 3 Northeast Dermatology Associates, Beverly, MA USA; 4 Stanford Department of Biomedical Data Science, Stanford, CA USA; 5 Stanford Department of Dermatology, Redwood City, CA USA; 6 Banner Health, Phoenix, AZ USA; 7 Wyss Institute for Bioinspired Engineering at Harvard, Boston, MA USA; 8 Duke University School of Medicine, Durham, NC USA

**Keywords:** Diagnosis, Diseases, Medical imaging, Skin manifestations

## Abstract

Although advances in deep learning systems for image-based medical diagnosis demonstrate their potential to augment clinical decision-making, the effectiveness of physician–machine partnerships remains an open question, in part because physicians and algorithms are both susceptible to systematic errors, especially for diagnosis of underrepresented populations. Here we present results from a large-scale digital experiment involving board-certified dermatologists (*n* = 389) and primary-care physicians (*n* = 459) from 39 countries to evaluate the accuracy of diagnoses submitted by physicians in a store-and-forward teledermatology simulation. In this experiment, physicians were presented with 364 images spanning 46 skin diseases and asked to submit up to four differential diagnoses. Specialists and generalists achieved diagnostic accuracies of 38% and 19%, respectively, but both specialists and generalists were four percentage points less accurate for the diagnosis of images of dark skin as compared to light skin. Fair deep learning system decision support improved the diagnostic accuracy of both specialists and generalists by more than 33%, but exacerbated the gap in the diagnostic accuracy of generalists across skin tones. These results demonstrate that well-designed physician–machine partnerships can enhance the diagnostic accuracy of physicians, illustrating that success in improving overall diagnostic accuracy does not necessarily address bias.

## Abstract (web summary)

In a large-scale study involving 389 board-certified dermatologists and 459 primary-care physicians from 39 countries, the impact of a deep learning-aided decision support system on physicians’ diagnostic accuracy was tested across 46 skin diseases and for both light and dark skin tones.

## Main

The future of machine learning in medicine is unlikely to involve substituting machines for physicians, but instead will involve physician–machine partnerships where domain-specific interfaces built on top of machine learning models may support clinical expertise in providing more accurate diagnoses for patients^1–9^. However, an emerging literature on human and artificial intelligence (AI) collaboration reveals that physician–machine partnerships are not guaranteed to be better than either physicians or machines alone^10–14^. In particular, experts may have trouble recognizing when to override or defer to algorithmic advice, which may be systematically biased in ways unknown to the expert^15^. Initial research in store-and-forward teledermatology suggests clinical decision support based on a deep learning system (DLS) can improve diagnostic accuracy by generalists^1^, but open questions remain about how physician–machine partnerships perform across levels of physician expertise and across underrepresented populations^16^.

Racial bias in medicine is well documented^17–21^. In dermatology there is a lack of representation of diverse skin tones that permeates textbooks^22,23^, residency programs^24^, dermatology research^25^, non-specialists’ diagnostic accuracy^26,27^ and training data for machine learning algorithms^28^. Although deep learning models show promise for enhancing clinical decision-making in dermatology^29,30^, algorithmic audits of deep learning models for dermatology reveal that these applied models often exhibit systematic errors on subsets of the data, especially on dark skin^31,32^. Recent research in machine learning applied to dermatology has focused on increasing the transparency in large-scale dermatology image datasets by annotating images with the estimated Fitzpatrick skin type (FST)^33^, developing new datasets with a focus on diversity^32^ and creating synthetic images with diffusion models^34^. These solutions can address some of the current issues of transparency and performance disparities^35^, but an open question remains of how accurately specialist and generalist physicians diagnose skin disease across skin tones in a store-and-forward teledermatology context, as well as how a physician–machine partnership may help to reduce (or possibly exacerbate) any potential differences in diagnostic accuracy across skin tones.

Methods from digital experiments in social sciences can be used for evaluating the accuracy and bias in medical decision-making and human–computer interactions. Similarly to how crowdworkers on MTurk enabled the transformation of experimentation in social and behavioral science a decade ago^36,37^, physician platforms offer an opportunity to recruit large numbers of physicians for surveys and diagnostic accuracy experiments^38^. We recruited a large number of physician participants by paying a nominal fee and designing the experiment to be a fun learning experience drawing on insights from gamified behavioral experiments^39^. In addition, we followed guidance from integrative experimentation^40^ and identified a reproducible experimental design space that covers the following dimensions: skin diseases, skin tones, physician expertise, physician–machine partnerships, clinical decision support accuracy, and user interaction designs. Our experiment focuses on measuring diagnostic accuracy with and without AI assistance across light and dark skin, and follows methods from algorithmic auditing^41^, which serves as a useful tool for systematically evaluating errors, exposing bias, and promoting transparency in machine learning algorithms^42^. We also build on recent work in diagnosing physician error^43,44^ to demonstrate that diagnostic accuracy experiments can offer insights into the performance of physicians and physician–machine partnerships.

## Results

### Study design

**Fig. 1:**
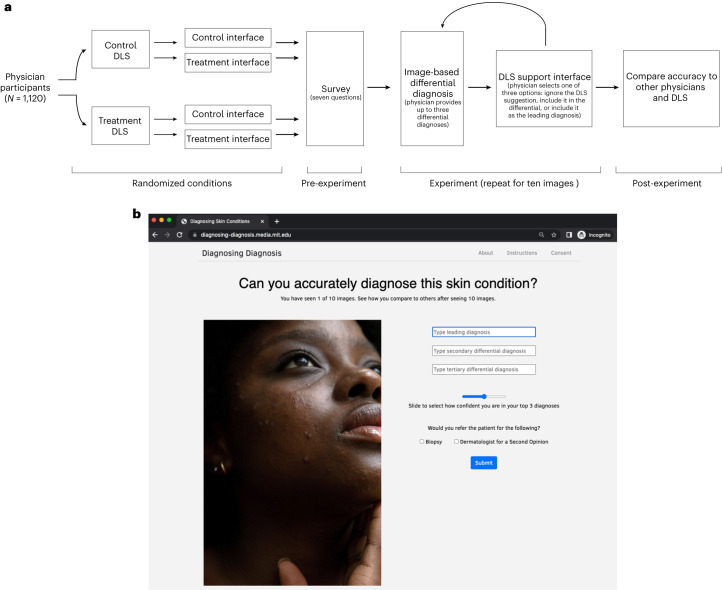
Experimental design flowchart and user interface. **a**, Flowchart describing the experimental design, including the number of participants, the randomly assigned conditions, the pre-experiment survey, the experiment, and the final stage, in which participants can see how they compare to other participants. **b**, Screenshot of the user interface for the image-based differential diagnosis portion of the experiment. Panel **b** reproduced with permission from Refinery29.

We designed a custom, digital experiment to evaluate physicians’ diagnostic accuracy on images of inflammatory-appearing skin diseases. This image-based experimental set-up mimics store-and-forward teledermatology and the types of patient images physicians are sent through electronic health record messaging systems, which often have minimal clinical context. We curated 364 images of 46 skin diseases. The vast majority of images (78%) depict the following eight main diseases, with at least 29 images for each disease: atopic dermatitis, cutaneous T-cell lymphoma (CTCL), dermatomyositis, lichen planus, Lyme disease, pityriasis rosea, pityriasis rubra pilaris, and secondary syphilis. The selected images represent a near uniform distribution across skin tones as measured by estimated FST. We hosted these images in an image-only, simulated store-and-forward experiment (outlined in Fig. 1), a setting that limits the amount of information available to the physician relative to the information available in an in-person clinical visit. Supplementary Figs. 1–5 provide additional screenshots of the experiment’s user interface.

The experiment begins with the randomized assignment of participants to two sets of conditions: two versions of the DLS and two interfaces for clinical decision support. The control DLS is a neural network architecture trained to classify nine classes (the eight main diseases and another class to represent all other diseases), has a top-1 accuracy of 47%, and is a fair classifier in the sense that accuracy is highly similar across FSTs. The treatment DLS is a Wizard of Oz classifier—a synthetically enhanced version of the control DLS, where we randomly re-assign 65% of wrong classifications to be correct classifications, resulting in 84% top-1 accuracy. The treatment DLS is designed to anticipate future DLS systems that may be substantially more accurate than today’s leading systems. The goal of this experimental set-up is not to assess the DLSs themselves, but rather to understand the impact of the DLSs on human decision-making. The control clinical decision support interface consists of three buttons in the following order: ‘Update my top prediction with [disease]’, ‘Update my differential to include [disease]’ and ‘Keep my differential’. The treatment interface consists of the same three buttons in reverse order, as shown in Supplementary Fig. 4. For full details of the DLS and the interface for clinical decision support, see the DLS development section in the Methods.

The experiment began by presenting participants with seven pre-survey questions, instructions, and the diagnostic accuracy task, where we ask participants to provide a differential diagnosis of up to three diseases (Supplementary Figs. 1 and 2 and Fig. 1 provide screenshots of the experimental interface). Next, we presented physicians with clinical decision support and asked them to decide whether or not to include the suggested diagnosis in their differential (Supplementary Fig. 4). In this experiment, we motivated participant engagement by informing them on the reference disease after each trial and displaying their overall performance after ten trials, which allowed physicians to learn about the content (for example, Which images correspond to which disease? How often is the decision support correct?) and themselves (for example, Did the participant diagnose the image correctly? How accurate is the participant compared to other specialists, generalists and the DLS?).

In the results presented here, we evaluate how accurately the specialist and generalist physicians diagnose images of inflammatory-appearing skin disease. We consider three measures of accuracy: top-1 accuracy (Does the participant’s leading diagnosis match the skin disease in the image?), top-3 accuracy (Do any of the participant’s initial three differential diagnoses match the skin disease in the image?) and top-4 accuracy (Do any of the participant’s initial three differential diagnoses or the decision support suggestion—if included by the participant—match the skin disease in the image?). We further evaluate how diagnostic accuracy differs across different skin tones in the images, as well as the physicians’ experience with different skin tones. Finally, we consider how DLS-based decision support influences diagnostic accuracy.

### Physician characteristics

In our digital, diagnostic accuracy experiment, we collected 14,261 differential diagnoses from 1,118 individuals on 364 images. This included 5,365 differentials from 389 board-certified dermatologists (BCDs), 1,691 differentials from 116 dermatology residents, 5,458 differentials from 459 individual primary-care physicians (PCPs) and 1,747 differentials from 154 other physicians. The first image shown in the experiment was an image of a woman with acne; this served as an attention check that physicians at all levels of expertise should be able to diagnose accurately. In total, 98% of BCDs, PCPs, and other physicians passed the attention check, and 96% of dermatology residents passed the attention check. Moreover, 76% of BCDs and PCPs, 73% of other physicians, and 72% of dermatology residents passed the attention check and provided differential diagnoses on at least ten images. After participants provided ten differential diagnoses, we thanked each participant for completing the experiment, revealed the aggregate performance of the other participants to the participant, and offered the participant an opportunity to continue diagnosing skin diseases in the experiment. Figure 1 provides an illustration of the experimental design.

In the sections on diagnostic accuracy, we focus our analysis on the first ten differentials provided by participants who passed the attention check and provided at least ten differentials. This includes 2,660 differentials from 296 BCDs, 747 differentials from 83 dermatology residents, 3,150 differentials from 350 PCPs and 1,015 differentials from 113 other physicians. Our results are robust to other selection criteria, such as only participants from the United States, participants who provided fewer than ten differentials, and all participants who pass the attention check (Supplementary Tables 1–4). This experiment included physicians living in 39 countries, half of whom live in the United States.

### Image quality

To ensure that the skin disease reference labels accurately represent the skin diseases in the images, we followed a five-step quality-control process with three BCDs, conducted a post hoc quality review, and evaluated the accuracy rates across image sources, as described in the Methods.

### Overall diagnostic accuracy

In the experiment, participants did not know which skin diseases would appear, and, as such, the accuracy of random guessing was near 0% (more details are provided in the Experimental interface subsection in the Methods). The top-3 accuracies of the BCDs, dermatology residents, PCPs and other physicians, as measured by any of their three differential diagnoses matching the reference label, were 38%, 36%, 19% and 18%, respectively, across all images in the experiment (excluding the attention check image) and 37%, 35%, 17% and 16%, respectively, across images of the eight main diseases in the experiment.

The top-1 accuracies, the accuracy of the leading diagnosis only, for the BCDs, dermatology residents, PCPs and other physicians were 27%, 24%, 14% and 13%, respectively, across all images in this experiment (excluding the attention check image) and 27%, 24%, 13% and 12%, respectively, across images of the eight main diseases in this experiment.

**Fig. 2:**
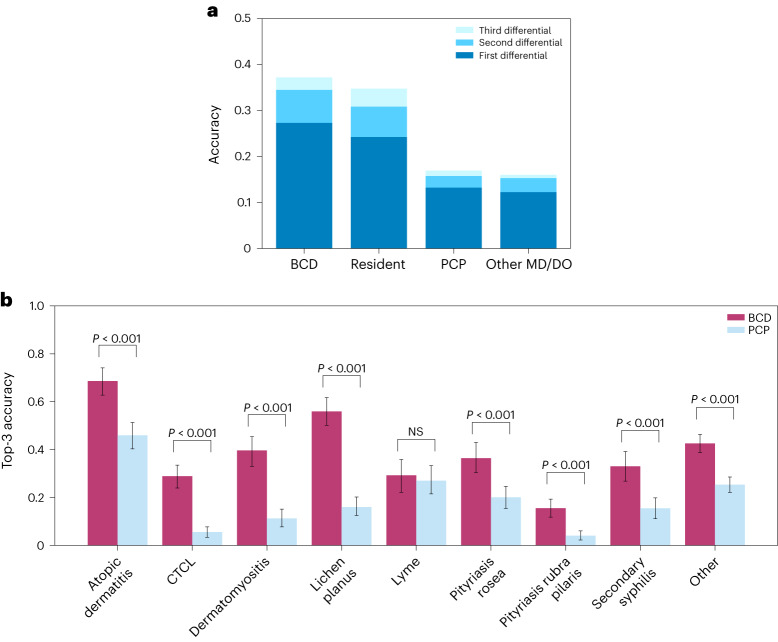
Diagnostic accuracy across skin diseases. **a**, Diagnostic accuracy of physician participants on the eight main skin diseases. Shades of blue indicate the diagnostic accuracy of the first, second and third differentials, respectively. ‘Resident’ refers strictly to dermatology residents. Other MD/DO refers to other physicians who have a doctor of medicine or doctor of osteopathic medicine degree. **b**, Top-3 diagnostic accuracy of BCDs (*N* = 296 physicians and *N* = 2,660 observations) and PCPs (*N* = 350 physicians and *N* = 3,150 observations) on each of the eight main skin diseases and the auxiliary 38 diseases, which are aggregated in the ‘Other’ category. All observations are represented as 1 or 0 for whether the submitted diagnoses match the consensus label or not. *P* values are calculated with a two-sided *t*-test. NS (not significant) indicates *P* > 0.05. Error bars represent the 95% confidence interval of the true mean.

Figure 2a presents the mean diagnostic accuracies of the participants split by their primary, secondary and tertiary diagnoses for images of the eight main diseases in this experiment.

Figure 2b presents the top-3 accuracies of the BCDs’ and PCPs’ full differential diagnoses across the eight main diseases and a category labeled ‘Other’, which aggregates the auxiliary 38 skin diseases into a single category. The BCDs significantly outperformed the PCPs at visually diagnosing skin diseases from images across seven of the eight skin diseases and the Other category. Extended Data Tables 1 and 2 show confusion matrices for how the consensus labels match the leading diagnoses of the BCDs and PCPs, respectively.

**Extended Data Fig. 1:**
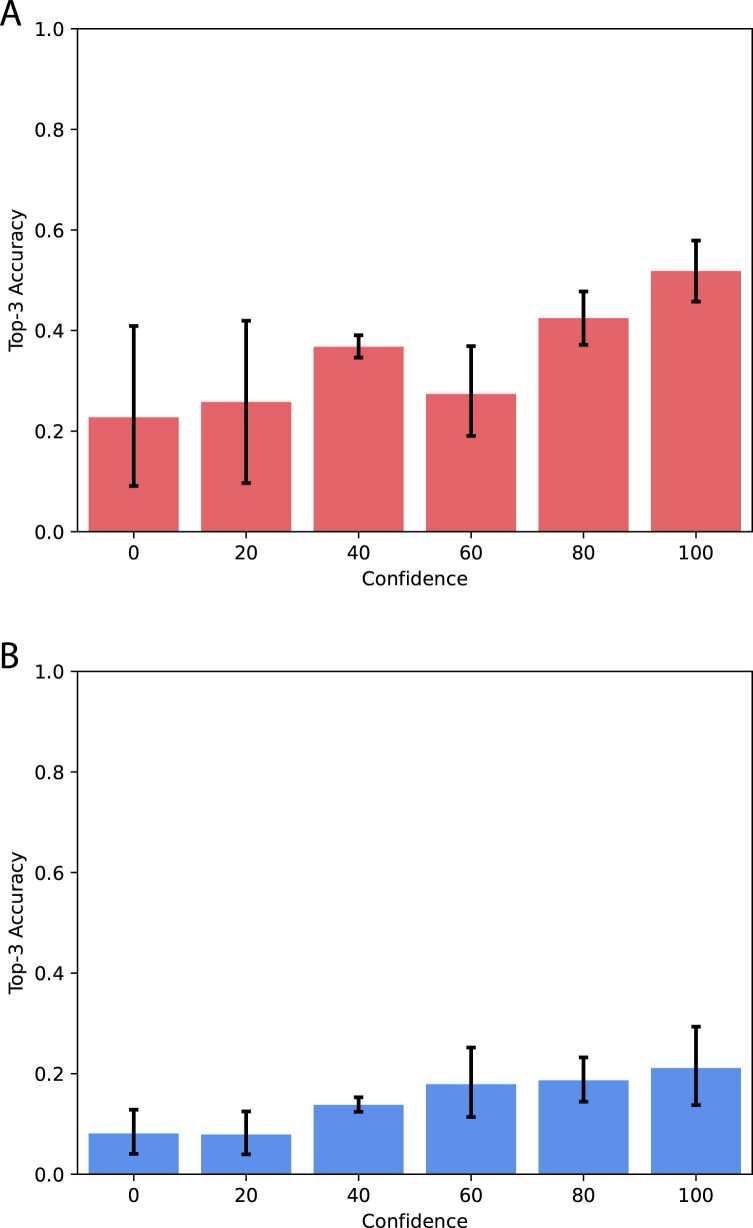
Diagnostic Accuracy by Reported Confidence. A. BCD (N = 296 physicians and 2660 observations) top-3 accuracy by reported confidence. B. PCP (N = 350 physicians and 3150 observations) top-3 accuracy by reported confidence. The measure of center for error bars is the mean accuracy within the following confidence bands: 0–10, 15–25, 30–50, 55–65, 70–85, 90–100. Participants reported confidence with numbers divisible by 5 between 0 and 100. The error bars represent 95% confidence intervals and the confidence interval for the interval that includes 50% is particularly small in both graphs because 71% of BCDs and 70% of PCPs did not change their confidence level from the default of 50%.

We found that the majority of BCDs and PCPs chose to respond with a default confidence of 50%. For participants who did not choose the default confidence, we found that top-1 and top-3 accuracies are positively correlated with confidence for the BCDs and PCPs, with Pearson correlation coefficients between 0.14 and 0.17. In Extended Data Fig. 1, we show participant accuracy by reported confidence.

We found that the most common leading diagnosis for each image by BCDs and PCPs is correct in 48% and 33% of observations, respectively. At least one BCD identified the reference label in their differential diagnosis in 77% of images, and at least one PCP identified the reference label in their differential diagnosis in 58% of images. After seeing a correct DLS prediction, at least one BCD included the reference label in their differential diagnosis in 98% of images.

### Diagnostic accuracy and clinical decision-making across light and dark skin

Across all images, we found that skin diseases in dark skin (estimated FST 5 and 6) are diagnosed less accurately than skin diseases in light skin (estimated FST 1–4). Across all participants, we found the top-1 and top-3 accuracies for skin diseases in dark skin to be four percentage points (*P* < 0.001 and *P* = 0.001, respectively) lower than for skin diseases in light skin. All statistical comparisons in this Article are based on ordinary least-squares regression with robust standard errors clustered at the participant level unless otherwise noted. When we examined the physician types separately, we found the top-1 accuracies of BCDs, dermatology residents, PCPs and other physicians to be lower by five percentage points (*P* = 0.011), five percentage points (*P* = 0.114), three percentage points (*P* = 0.006) and five percentage points (*P* = 0.012) for images of dark skin than light skin, respectively. Similarly, the top-3 diagnostic accuracies of BCDs, dermatology residents, PCPs and other physicians were lower by three percentage points (*P* = 0.117), five percentage points (*P* = 0.113), four percentage points (*P* = 0.008) and four percentage points (*P* = 0.092) for images of dark skin than light skin, respectively. We found qualitatively similar results in a series of robustness checks including only participants who live in the United States, participants who provided fewer than ten responses, and all responses from all participants who passed the attention check revealed similar results (Supplementary Tables 1, 2, 3 and 4).

**Fig. 3:**
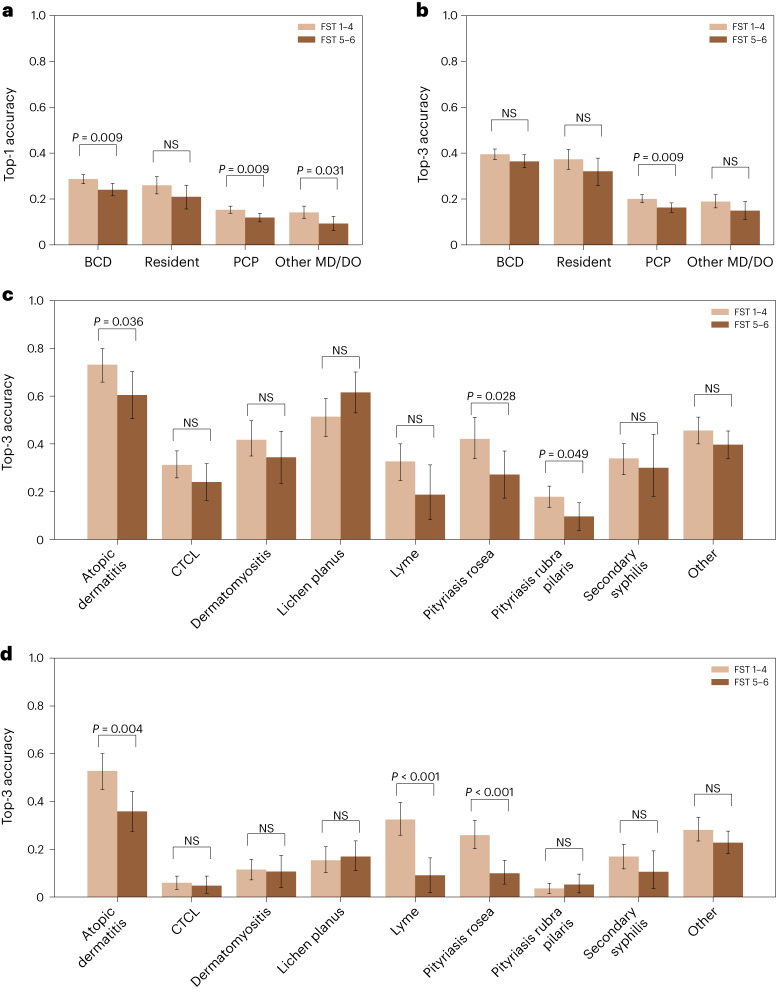
Diagnostic accuracy across FST. **a**, Top-1 diagnostic accuracies of BCDs (*N* = 296 physicians and 2,660 observations), dermatology residents (*N* = 83 residents and 747 observations), PCPs (*N* = 350 physicians and 3,150 observations) and other physicians (*N* = 113 physicians and 1,015 observations) across estimated FSTs on the eight main diseases. *P* values are calculated with a two-sided *t*-test. NS, *P* > 0.05. Error bars represent the 95% confidence interval of the true mean. **b**, Top-3 diagnostic accuracies of physician participants across estimated FSTs on the eight main diseases. **c**, Top-3 diagnostic accuracy of BCDs across skin diseases and FSTs (Supplementary Fig. 7 provides a fine-grained breakdown of accuracy by FST). **d**, Top-3 diagnostic accuracy of PCPs across skin diseases and FSTs.

**Extended Data Fig. 2:**
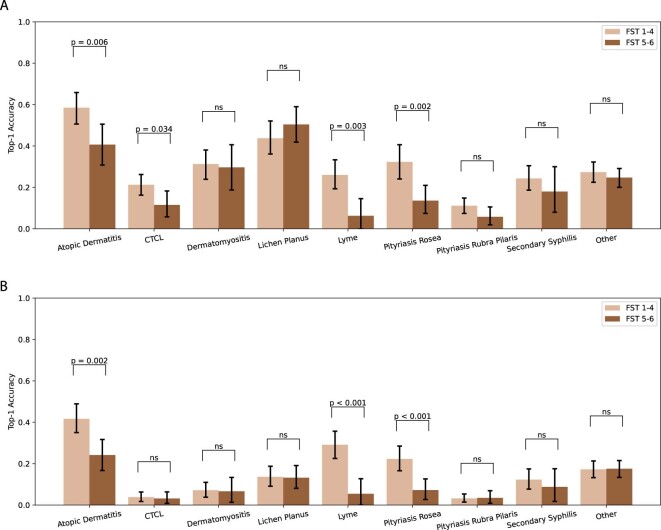
Top-1 Diagnostic Accuracy across Skin Diseases and Skin Tones. A. BCD (N = 296 physicians and 2660 observations) top-1 accuracy across skin diseases and tones. B. PCPs (N = 350 physicians and 3150 observations) top-1 accuracy across skin diseases and tones. P-values are calculated with a two-sided t-test and ns indicates that the p-value is greater than 0.05. The error bars represent the 95% confidence interval of the true mean.

Fig. 3c,d presents the top-3 diagnostic accuracy across skin diseases for BCDs and PCPs, respectively (Extended Data Fig. 2 presents the top-1 diagnostic accuracy across skin diseases). BCDs diagnosed seven out of eight skin diseases and the Other category with higher accuracy for light skin than dark skin images. The only skin disease in which BCDs were more accurate on dark skin than light skin is lichen planus. We do not find statistically significant differences in top-3 accuracy across skin tones across individual skin diseases for BCDs, but we find statistically significant differences in BCDs’ top-1 accuracy across light and dark skin images in four diseases—atopic dermatitis, Lyme disease, pityriasis rosea and CTCL—18 percentage points (*P* = 0.007), 20 percentage points (*P* < 0.001), 19 percentage points (*P* = 0.001) and 10 percentage points (*P* = 0.009) lower on dark skin, respectively (these p-values are based on ordinary least-squares regressions with robust standard errors clustered at the participant level, which are nearly but not exactly the same as the p-values from the t-test presented in Extended Data Figure 2). We find statistically significant and large differences in the top-3 and top-1 diagnostic accuracies of PCPs across light and dark skin images in three diseases: atopic dermatitis, Lyme disease and pityriasis rosea, respectively.

**Extended Data Fig. 3:**
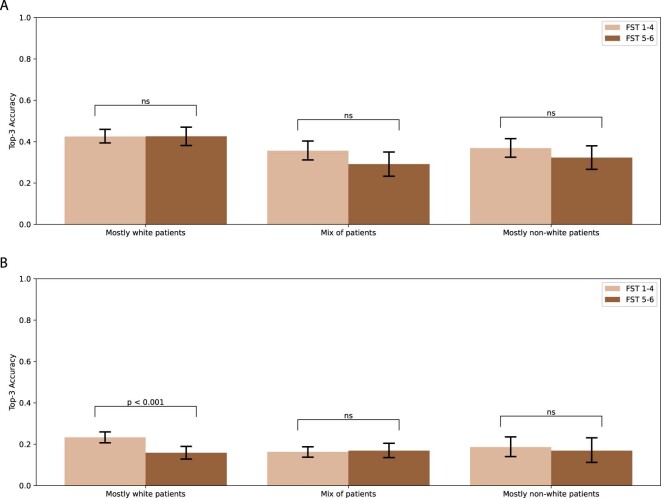
Diagnostic Accuracy by Providers’ Experience with Diverse Patients. A. BCDs’ (N = 291 physicians and 2615 observations) top-3 accuracy B. PCPs’ (N = 343 physicians and 3087 observations) top-1 diagnosis on each of the 8 main skin diseases in this experiment. The categories on the x-axis are based on self-reported responses to “How would you describe the distribution of your patients’ skin color?” We exclude BCDs and PCPs who respond “Not Applicable.” P-values are calculated with a two-sided t-test and ns indicates that the p-value is greater than 0.05. The error bars represent the 95% confidence interval of the true mean.

We found that accuracy disparities across skin tones are moderated by the diversity of patients seen by PCPs and PCP training. In particular, we found that PCPs who report seeing mostly or all white patients are seven percentage points (*P* = 0.009) less accurate (top-3) on dark skin images than light skin images. We did not find statistically significant differences for BCDs based on self-reported patient diversity (a bar chart is provided in Extended Data Fig. 3). Likewise, we found that PCPs who reported sufficient training were five percentage points (*P* = 0.079) more accurate (top-3) than PCPs who reported insufficient training on images of dark skin than light skin. We did not find statistically significant differences in BCDs’ top-1 or top-3 accuracies with respect to their self-reported sufficient training on dark skin. Similarly, we did not find statistically significant differences in BCDs’ or PCPs’ top-1 or top-3 accuracies with respect to their years of experience or self-reported difficulty with white patients relative to non-white patients.

**Fig. 4:**
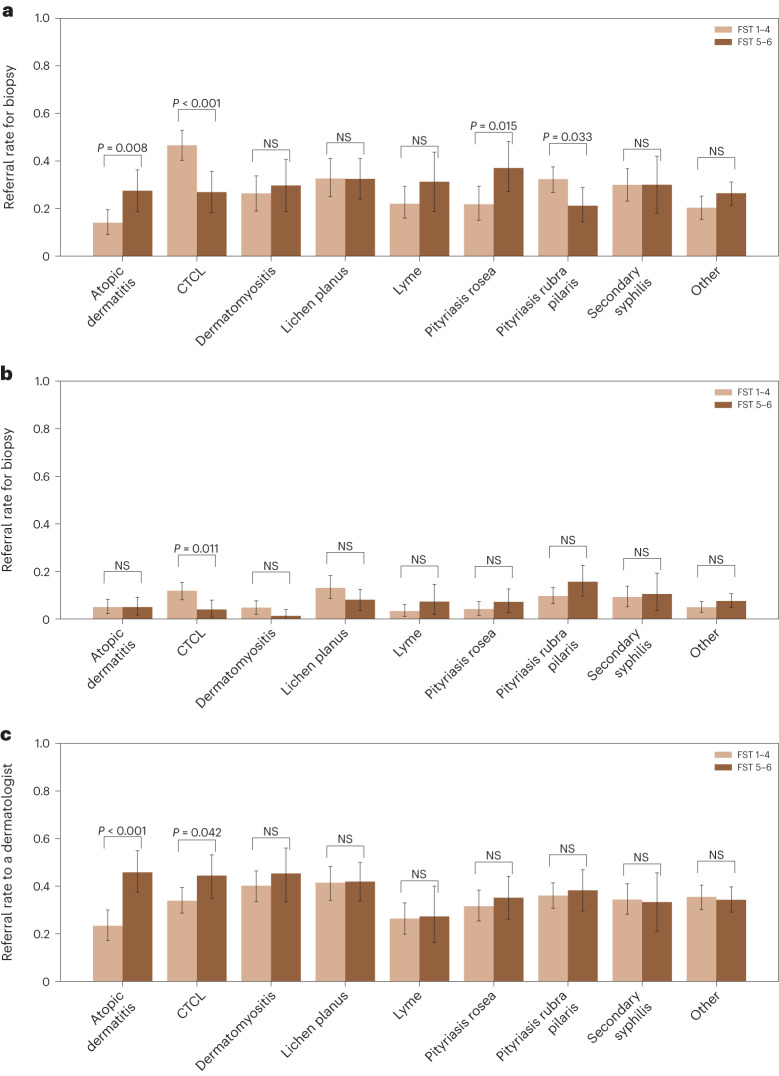
Referral rates for biopsy and a specialist’s opinion across FST. **a**, Biopsy referral rate of BCDs (*N* = 296 physicians and 2,660 observations) across skin diseases and tones. *P* values are calculated with a two-sided *t*-test. NS, *P* *>* 0.05. Error bars represent the 95% confidence interval of the true mean. **b**, Biopsy referral rate of PCPs (*N* = 350 physicians and 3,150 observations) across skin diseases and tones. **c**, PCP referral rate to a dermatologist (*N* = 350 physicians and 3,150 observations) across skin diseases and tones.

We also asked participants whether they would refer a patient for biopsy and asked non-BCDs whether they would refer the patient for a second opinion by the dermatologist. Figure 4 presents biopsy and dermatologist second opinion referral rates. BCDs indicate they would refer a patient for biopsy in 28% of observations. In contrast, PCPs indicate they would refer a patient for biopsy in only 7% of observations and refer a patient to a dermatologist in 28% of observations. We found that BCDs refer common, non-life-threatening diseases (atopic dermatitis (*P* = 0.008) and pityriasis rosea (*P* = 0.015) for biopsy at significantly higher rates for dark skin than light skin, and refer pityriasis rubra pilaris (a rare disease; *P* = 0.033) and CTCL (a rare and potentially life-threatening diseases; *P* = 0.001) for biopsy at significantly lower rates for dark skin than light skin. We did not find statistically significant differences for BCDs’ biopsy referral rates across skin tones in other skin diseases, and we did not find statistically significant differences for PCPs’ biopsy referral rates except for CTCL (*P* = 0.011). We found that PCPs are 4.4 percentage points (*P* = 0.012) more likely to refer patients with dark skin than patients with light skin for a dermatologist second opinion.

### DLS assistance

**Extended Data Fig. 4:**
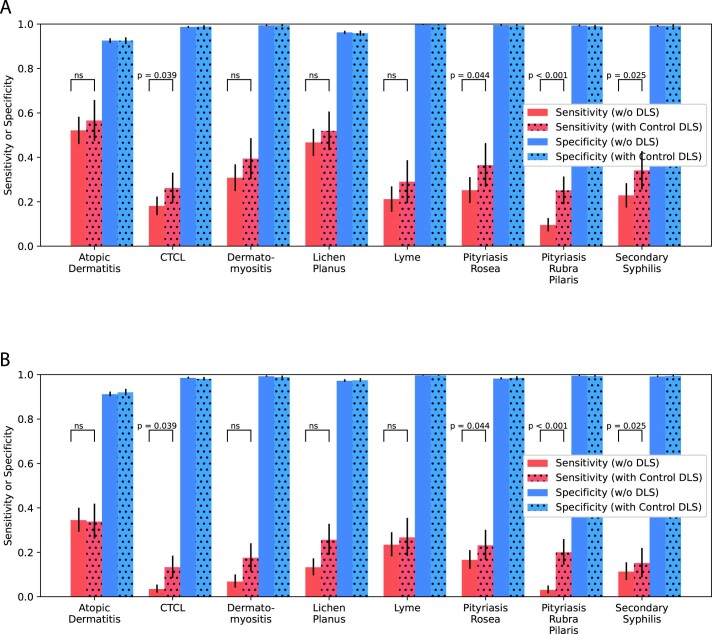
Sensitivity and specificity of physician’s diagnostic accuracy with and without DLS assistance. A. BCDs’ (N = 296 physicians and 2079 observations) top-1 sensitivity and specificity on each of the 8 main skin diseases in this experiment. B. PCPs’ (N = 350 physicians and 2496 observations) top-1 sensitivity and specificity on each of the 8 main skin diseases in this experiment. P-values are calculated with a two-sided t-test and ns indicates that the p-value is greater than 0.05.

We found that DLS decision support significantly increases diagnostic accuracy, while leading to the inclusion of relatively few incorrect diagnoses. With access to suggestions from the control DLS, BCDs’ and PCPs’ top-1 accuracy on the main eight diseases increases from 27% to 36% (*P* < 0.001, *t*-test) and from 13% to 22% (*P* < 0.001, *t*-test), respectively. In other words, BCDs with DLS assistance are 33% more accurate in their leading diagnoses, and PCPs with DLS assistance are 69% more accurate in their leading diagnoses. More specifically, we found that BCD’s sensitivity for diagnosing CTCL increases from 18% to 26% (*P* = 0.039, *t*-test) with the control DLS and 31% (*P* = 0.001, *t*-test) with the treatment DLS, whereas the BCDs’ specificity remained generally constant at 99% without DLS assistance and 99% with the control DLS or with the treatment DLS. Extended Data Fig. 4 reports the sensitivity and specificity of BCDs and PCPs with and without access to the control DLS across each of the eight main skin diseases in this experiment.

**Fig. 5:**
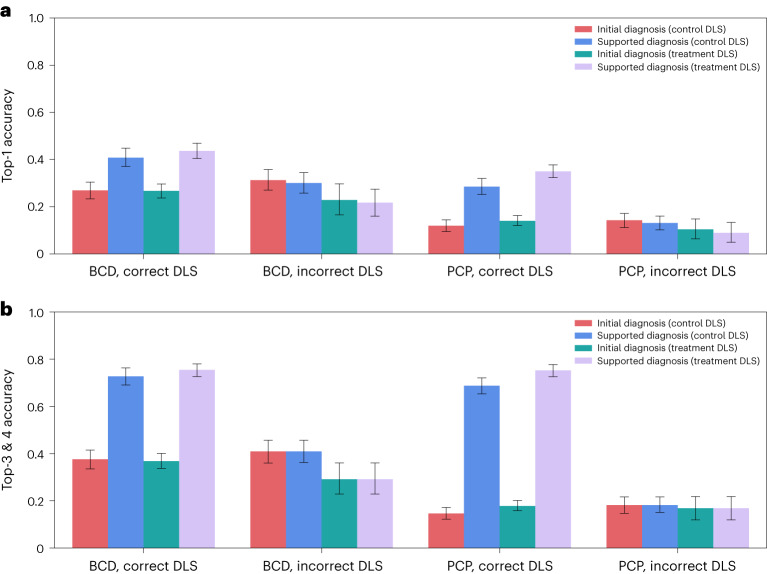
Diagnostic accuracy of physicians with and without DLS assistance. **a**, Top-1 accuracy of physicians before and after seeing either the control or treatment DLS suggestion. **b**, Top-3 and top-4 accuracies of physicians before and after seeing the control or treatment DLS suggestion (BCD: *N* = 296 physicians and 2,079 observations; PCP: *N* = 350 physicians and 2,496 observations). Error bars represent the 95% confidence interval of the true mean.

We found even larger accuracy gains when moving from top-3 accuracy without DLS support to top-4 accuracy with control DLS support on the main eight diseases: the BCDs’ accuracy increased from 37% to 60%, and the PCPs’ accuracy increased from 17% to 47%. Alternatively, if we replaced participants’ third differential diagnosis with the DLS suggestions that participants include in their differential diagnoses, we found that the BCDs’ top-3 accuracy was 59% and the PCPs’ top-3 accuracy was 46%. For simplicity and conciseness, throughout this Article we report top-4 accuracy when including DLS suggestions rather than top-3 accuracy with replacement of the third differential diagnosis. Figure 5 shows physicians’ top-1 accuracy (Fig. 5a) and top-3 and top-4 accuracies (Fig. 5b) before and after they see the DLS-based suggestions.

When we restricted our analysis to the 236 images on which the control and treatment DLSs make the same predictions, we found that the BCDs and PCPs update their differential in 40% and 54% of diagnoses with the control DLS and in 47% and 61% of diagnoses with the treatment DLS, and these differences are significant at the *P* = 0.009 and *P* = 0.001 levels, respectively.

On images where the DLS made an incorrect suggestion, we found minimal effects on BCDs’ and PCPs’ top-1 accuracies, which both decrease by 1.2 percentage points (*P* = 0.517 and 0.312, respectively, *t*-test). In instances where the DLS provided an incorrect suggestion, we found that the BCDs and PCPs overrode their correct leading diagnosis with an incorrect suggestion in fewer than 2% of observations. In contrast, when the decision support provided an incorrect suggestion and the BCDs’ and PCPs’ three differential diagnoses were all incorrect, we found that the BCDs and PCPs included incorrect suggestions as leading diagnoses in 10% and 14% of observations, respectively. The BCDs’ top-4 accuracy with decision support included 1.58 incorrect diagnoses per observation and top-3, top-2 and top-1 accuracies without decision support included 1.40, 1.05 and 0.59 incorrect diagnoses per image, respectively. In contrast, the PCPs’ top-4 accuracy with the decision support included 1.72 incorrect diagnoses per observation, whereas the top-3, top-2 and top-1 accuracies without decision support included 1.55, 1.26 and 0.82 incorrect diagnoses per image, respectively.

With respect to top-1 accuracy, we found that the BCDs without decision support were five percentage points (*P* < 0.001, *t*-test) more accurate than PCPs with control DLS decision support, but four percentage points (*P* = 0.022, *t*-test) less accurate than PCPs with treatment DLS decision support.

**Fig. 6:**
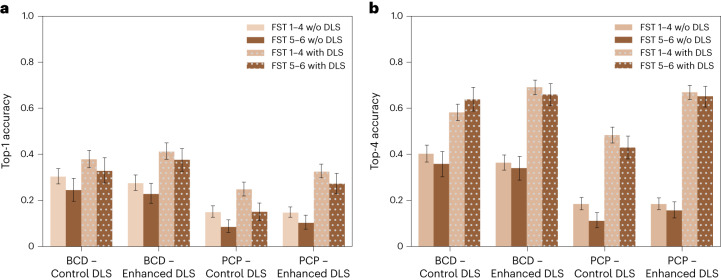
Diagnostic accuracy of physicians with and without DLS assistance across FSTs. **a**,**b**, Top-1 (**a**) and top-4 (**b**) accuracies of the physicians (*N* = 296 BCD physicians and 2,079 BCD observations; *N* = 350 PCP physicians and 3,150 PCP observations) and physician–machine partnerships across light and dark skin tones for the eight main conditions. The dotted bars indicate results for physician–machine partnerships. Error bars represent the 95% confidence interval of the true mean.

Extended Data Table 3 presents ordinary least-square regressions on diagnostic accuracy based on the following independent variables: physician expertise, skin tone in an image, DLS suggestions and interactions between these variables. This regression table, where we focus on BCDs and PCPs, presents top-1 accuracy in the first column and top-4 accuracy in the second column. For top-1 accuracy, BCDs are 13 percentage points more accurate than PCPs (*P* < 0.001), participants are three percentage points less accurate on images of dark skin (*P* = 0.006), the DLS suggestions leads to eight percentage points higher performance overall (*P* < 0.001), and the treatment DLS leads to an additional eight percentage point increase in accuracy (*P* = 0.002). Likewise, we find the control DLS suggestion exacerbates the accuracy disparities in PCPs’ diagnoses by five percentage points (*P* = 0.008 and 0.048, respectively, for top-1 and top-4 accuracies), but we do not find statistically significant evidence that accuracy disparities increase for BCDs. The three-way interaction between BCDs, dark skin and the DLS suggestion shows that the DLS suggestions on dark skin lead to a marginal four and eight percentage point increase in top-1 and top-4 accuracies (*P* = 0.227 and *P* = 0.034), respectively. As a result, in Fig. 6 we continue to find statistically significant evidence for accuracy disparities for PCPs but not for BCDs. In Extended Data Table 4, we present the same ordinary least-square regressions, where we also include interactions with the control and treatment user interface assignments, and we do not find significant interaction effects.

### User interaction design

We did not find any statistically significant differences between the control and treatment conditions in relation to whether participants chose to ignore or include suggestions in their differential diagnoses. However, we found a significant effect of the order of options on participants’ choice to update their leading diagnosis with the suggestion versus updating their differential diagnosis to include the suggestion. Specifically, we found the treatment condition (with ‘Update my top prediction’ on the bottom) leads participants to select ‘Update my differential’ nine percentage points (*P* < 0.001) more often and ‘Update my top prediction’ nine percentage points (*P* < 0.001) less often. Extended Data Table 5 presents regressions showing average treatment effects of the interface randomization on participants’ choices to update their differential diagnoses. As a consequence, we found BCD–machine partnerships and PCP–machine partnerships assigned to the treatment condition are 12 percentage points (*P* < 0.001) and seven percentage points (*P* = 0.011) lower, respectively, in top-1 accuracy than the partnerships assigned to the control condition.

## Discussion

As we move towards a future where algorithms and physicians work collaboratively, it is important to understand the baseline bias of physicians and how algorithms will influence those biases. Using skin disease as a case study, we assessed the baseline accuracy of specialist and generalist physicians in diagnosing skin disease across skin tones in a simulated store-and-forward teledermatology setting. The eight main skin diseases in this experiment often present differently depending on a patient’s skin tone. For example, the classic presentation of Lyme disease as a red or pink bulls-eye rash in light skin may appear brown, black, purple or even off-white in darker skin^45^. Similarly, atopic dermatitis appears red in lighter skin and purple in darker skin and often involves more dryness, lichenification and hyper- and hypopigmentation in darker skin tones. In contrast, the classic presentation of lichen planus involves a violet hue, which is more common in dark skin than light skin, where it can also present as pink or red. In addition, some of these skin diseases appear more often in prevalence rate data in white people than black people (for example, Lyme disease^46^) and vice versa (for example atopic dermatitis^47^). However, prevalence rate data are impacted by diagnostic biases and access to care and can be misleading regarding the true prevalence rates.

As a baseline, in this experiment we found the top-3 diagnostic accuracy of BCDs to be 38% and of PCPs to be 19% (and 42% and 19% for United States-based BCDs and PCPs, respectively) on images of inflammatory-appearing skin diseases. These results match past research demonstrating that specialists significantly outperform generalists at skin-disease diagnosis, but show lower diagnostic accuracy than past studies with different experimental set-ups^48–52^. Given our quality-control protocol, the post hoc qualitative review and the similar error rates across sources (described in the Methods), these results cannot be explained by mislabeled images. Instead, our results, which may seem surprising due to the low accuracy rate of specialists on inflammatory-appearing skin diseases, are best explained by the difficulty of diagnosing these diseases with free-response (as opposed to multiple choice) answers and the differences between this store-and-forward teledermatology setting (where a physician has access to only a single image) and an in-person patient interaction (where a physician has access to much more information such as better lighting, better field of view, and the ability to inquire about a patient’s symptoms, lifestyle, clinical history, family history and more). Although in-person clinical visits are the gold standard, image-based store-and-forward teledermatology has gained traction in triage^53^ and can serve as a use case for looking at baseline physician accuracy and physician–AI interaction. Moreover, physicians often receive patient messages with photographs attached, with minimal context, and are asked to make a determination of whether the patient should come in for a clinical visit. The results from this experiment reveal the limits to diagnosing skin disease from a single image and highlight the importance of considering information beyond visual features.

We find that the diagnostic accuracy of specialists and generalists is lower on images of dark skin than light skin. Specifically, when comparing participants’ three differential diagnoses to the quality-controlled skin disease reference labels, we found that BCDs and PCPs are four percentage points more accurate on images of light skin (FST 1–4) than dark skin (FST 5 and 6). These differences in accuracy across skin tones are statistically significant. Given BCDs’ and PCPs’ accuracy rates of 38% and 19%, respectively, images of dark skin are diagnosed 10% less accurately than images of light skin by BCDs and 22% less accurately by PCPs. These results contribute to an emerging literature on diagnostic accuracy disparities across patient skin tones^26,27^ and present evidence that the diagnostic accuracy of medical professionals on images of dark skin is lower than on images of light skin.

Furthermore, we have found differences in how often BCDs and PCPs refer patients with light and dark skin for biopsy. Specifically, for CTCL (a life-threatening disease), we found that both BCDs and PCPs report that they would refer patients for biopsy significantly more often in light skin than dark skin. Moreover, for the common skin diseases atopic dermatitis and pityriasis rosea, we found that BCDs report they would refer patients for biopsy more often in dark skin than light skin, which creates an unnecessary overburden on patients with dark skin.

By first establishing a benchmark for the diagnostic accuracy of physicians across skin tones in this well-defined task, we could evaluate DLS assistance by comparing the baseline benchmark to the diagnostic accuracy of physician–machine partnerships. We found that DLS-based decision support increases top-1 diagnostic accuracy by 33% for BCDs and 69% for PCPs. This translates into improved sensitivity in diagnosing specific skin diseases with minimal effects on specificity; for example, we found that specialists’ sensitivity for diagnosing CTCL increased by 44% with control DLS assistance and 72% with treatment DLS assistance, whereas the specialists’ specificity remained constant. From a clinical perspective, these large increases in overall accuracy by physician–machine partnerships are relevant for informing the design of diagnostic procedures to improve triage and reduce delayed and missed diagnoses. We note that BCDs' top-1 accuracy without decision support remains higher than PCPs' top-1 accuracy with control decision support.

The physician–machine partnerships in the form of physicians interacting with decision support based on a DLS in this experiment led to minimal errors. We found that physicians rarely override their leading diagnosis when it is correct, but specialists and generalists can be influenced by the DLS to include incorrect diagnoses in their differential diagnosis. We found that a minor design choice—the order of whether to include a DLS suggestion as a leading diagnosis, one of the diagnoses, or ignore the suggestion—significantly influences participants’ choices. This indicates that, in addition to the accuracy of the classifier, the presentation interface is an important consideration for human–AI interactions.

Although physician–machine partnerships improve overall diagnostic accuracy, we have found that the DLS-based decision support exacerbates non-specialists’ diagnostic accuracy disparities for light and dark skin. However, we did not find that the DLS significantly influences the diagnostic accuracy disparities of specialists. One potential explanation for the magnification of diagnostic accuracy disparities in generalists (despite the overall improved accuracy) may be related to the nature of the DLS prompting physicians to consider alternatives that they cannot rule out and generalists’ limited knowledge of what can and cannot be ruled out in dark skin. These results reveal the importance of human–AI testing in the intended use setting, because, in this experiment, the DLS, which does not exhibit disparate impact across skin tones, had a different impact on diagnostic accuracy disparities depending on whether the DLS was used to support generalists or specialists.

These results show that DLS assistance can significantly improve physicians’ diagnostic sensitivity while maintaining the same general level of specificity, but accuracy disparities can still increase, which raises the question of how to weigh accuracy gains against fairness and how differential performance across levels of expertise should be treated from a policy-making perspective.

This digital experiment for evaluating diagnostic accuracy resembles a store-and-forward teledermatology setting, but does not fully match a clinical evaluation in either teledermatology or an in-person examination. A single image contains substantially less information than an in-person interaction (or even a video call), which could include additional visual information (for example, adjustments in light and angle of view), a patient’s symptoms, clinical history, behavioral information and more. This Article serves as an assessment of physicians’ ‘know what’ on a very specific, constrained task where a physician has access to a single image, but not physicians’ ‘know how’^54^ of interacting with, caring for and listening to a patient, which is essential for diagnosing and intervening in a patient’s disease^55,56^.

Future work should consider diagnostic accuracy in clinical settings and further examine how DLS-based decision support compares to collective human intelligence-based decision support^57–60^. In the meantime, physicians should seek additional training in diagnosing dark skin diseases to avoid the potential for systematic misdiagnoses in clinical settings that may mirror the systematic differences found in diagnosing light and dark skin in this experiment.

## Methods

### Ethics approval

This research complies with all relevant ethical regulations. The Massachusetts Institute of Technology’s Committee on the Use of Humans as Experimental Subjects approved this study as Exempt Category 3—Benign Behavioral Intervention, which can be found under identification numbers E-2875 and E-3675. At the beginning of the experiment, all participants were presented with the following informed consent statement: ‘This is an MIT research project. We will first ask 7 brief survey questions. Then, we will show you images of skin conditions and ask you to try to diagnose the skin conditions. After you diagnose conditions in 10 images, we will show you how you perform relative to other healthcare providers. All submissions are collected anonymously for research purposes. For questions, please contact dermatology-diagnosis@mit.edu. Participation is voluntary’. The individual whose image appears in each figure has provided consent for their image to be published.

### Experimental interface

We designed and deployed a custom website at https://diagnosing-diagnosis.media.mit.edu to host the diagnostic accuracy experiment. On clicking the link to the website, participants are directed to the landing page, where we provide informed consent and ask several questions as shown in Supplementary Fig. 1. After participants fill out the survey, the website directs participants to instructions via a modal window, as shown in Supplementary Fig. 2. Specifically, we note that ‘the AI model is not perfectly accurate’, and we intentionally do not disclose the model’s accuracy. There are several reasons for this. First, we did not want to bias adherence to the model’s suggestions based on the model’s accuracy base rate. Second, we wanted to evaluate how often physician participants would accept suggestions for different base rates of accuracy (the control and treatment DLS) without explicitly sharing those base rates. Moreover, this maps to real-world situations where physicians may know an AI has been approved for a particular task but may not know the details on its accuracy with respect to the local context of their patients.

Once participants close the modal, they can begin the experiment, as shown in Supplementary Fig. 1. All participants see the same first image of a woman with acne, which serves as a relatively easy image to diagnose and a robustness check to confirm participants are participating seriously. Participants are asked ‘Can you accurately diagnose this skin condition?’, and they are informed how many images they have seen and that they will see how they compare to others after seeing ten images. Participants can provide up to three differential diagnoses, and the three text response forms display ‘Type leading diagnosis’, ‘Type secondary differential diagnosis’ and ‘Type tertiary differential diagnosis’. Participants can move a slider to provide how confident they are from 0% confident to 100% confident. In addition, participants are asked to check the boxes for whether they would refer the patient for a biopsy or a dermatologist for a second opinion. BCDs are not asked whether they would refer to a dermatologist because they are dermatologists.

When a participant begins to type their diagnosis in the free-response text boxes, predictive text appears as shown in Supplementary Fig. 3. We designed this experiment with free responses instead of multiple choice responses to maintain as much ecological validity to clinical practice as possible. Free response is more difficult than multiple choice for two main reasons. First, multiple choice enables correct answers via uninformed guessing, whereas free responses do not. Second, multiple choice primes the participant on what a particular disease might be, whereas free responses do not. We supported free responses with predictive text based on 445 possible diagnoses to promote standardized responses. These 445 diagnoses include the 46 skin diseases in this experiment, the 419 skin diseases in ref. ^30^, which have large overlap with the skin diseases in this experiment, and similar clinical terms for skin diseases. Three examples of similar clinical terms include atopic dermatitis and eczema, CTCL and mycosis fungoides, and Lyme disease and erythema migrans. The predictive text appears as a function of the first characters typed, and, to encourage participants to choose from the list, we attempted to include as many ways of writing diseases as possible (for example ‘erythema migrans (Lyme)’ and ‘lyme (erythema migrans)’ or ‘ctcl (cutaneous t-cell lymphoma)’ and ‘cutaneous t-cell lymphoma (ctcl).’

Once a participant clicks ‘submit’ (and assuming the participants’ differential diagnosis differs from the AI’s prediction), the website directs participants to a page showing the AI’s prediction. If the AI predicts ‘Other’, then we randomly select a disease from the 36 auxiliary diseases as the suggestion. Participants have three options: ‘Keep my differential’, ‘Update my differential to include [suggested disease]’ or ‘Update my top prediction with [suggested disease]’, as shown in Supplementary Fig. 4. Next (or if the participant’s differential matched the suggestion), the website directs participants to a page offering feedback on what the reference diagnosis is and what the most common incorrect diagnosis for this image was, as shown in Supplementary Fig. 5.

When participants click ‘Next Image’ on the feedback page, participants are redirected to a page that looks like Fig. 1 but with a different image, and the experiment repeats for as long as a participant is willing to participate. After a participant sees ten images, we show participants a bar graph showing how the diagnostic accuracy compares across the DLS, specialists and generalists.

### Clinical image curation

The experiment contains 364 images of 46 different skin diseases. The vast majority of images show eight relatively common skin diseases. There are 31 images of atopic dermatitis, 48 of CTCL, 34 of dermatomyositis, 30 of erythema migrans (Lyme disease), 32 of lichen planus, 33 of pityriasis rosea, 47 of pityriasis rubra pilaris and 29 of secondary syphilis. We decided to focus our analysis on these eight diseases based on three criteria: first, three practicing BCDs identified these diseases as the most likely diseases on which we may find accuracy disparities across patients’ skin tones; second, these diseases are relatively common; third, these diseases appear frequently enough in dermatology textbooks and dermatology image atlases such that we could select at least five images of the two darkest skin types after applying a quality-control review by BCDs. According to data from the All of Us research program, prevalence rates of the eight main diseases from most to least prevalent are atopic dermatitis (2.69%), Lyme disease (0.86%), lichen planus (0.53%), pityriasis rosea (0.36%), dermatomyositis (0.13%), secondary syphilis (0.10%), pityriasis rubra pilaris (0.02%) and CTCL (less than 0.01%)^61^. Literature reviews of the prevalence rates of each skin disease corroborate these prevalence rates from the All of Us research program within an order of magnitude^62–69^. We sourced the 284 images of the eight diseases based on 241 publicly available images online from dermatology atlases and search engines, 30 images from 14 textbooks, and 13 images from dermatologists’ slides and education material^70–94^. The number of images from each source is provided in Extended Data Table 6.

The remaining 80 images represent 38 skin diseases and are all drawn from the Fitzpatrick 17k dataset^31^, except for the attention check, which is sourced from a magazine article on inflammatory diseases in dark skin^95^. We included these additional diseases primarily to promote the ecological validity of the experiment. In particular, we designed this experiment such that participants do not know which skin diseases will appear in the experiment, and, as such, participants cannot simply treat this as a multiple-choice test. Beyond the eight diseases of direct interest, there are eight images of scleroderma, six of lupus erythematosus, six of acne, four of vitiligo, three of rosacea, three of tungiasis, three of urticaria pigmentosa, three of sarcoidosis, two of cheilitis, two of calcinosis cutis, two of allergic contact dermatitis, two of factitial dermatitis, two of fixed eruptions, two of granuloma annulare, two of keloid, two of keratosis pilaris, two of acanthosis nigricans, two of rhinophyma, two of necrobiosis lipoidica, two of tick bite, two of papilomatosis confluentes and reticulate, two of psoriasis, two of scabies, one of livedo reticularis, one of urticaria, one of Steven Johnson syndrome, one of statis edema, one of seborrheic dermatitis, one of erythema nodosum, one of erythema elevatum diutinum, one of lichen simplex, one of neurotic excoriations, one of hidradenitis, one of nematode infection, one of lichen amyloidosis and one of xanthomas.

We curated the images of skin diseases with the following five steps. First, we collected all images of the eight skin diseases from online sources and textbooks and the attention check image from an online magazine. Second, we annotated images with estimated FST labels. One BCD curated 351 of the highest-quality images of the eight diseases of interest for each of the six FSTs by dragging and dropping images into folders on their computer, specifying the skin disease and FST label. Due to a lack of images of secondary syphilis in light-skin instances and Lyme disease in dark skin, this first BCD supplemented the dataset with 11 images from their educational materials. Third, a second BCD reviewed the initially selected images and identified 66 images as low in quality due to image resolution or with questions about the diagnostic label. We removed these 66 images from the dataset to leave 285 images of the eight diseases remaining. Fourth, we added 79 images of 38 skin diseases from the Fitzpatrick 17k dataset that have been reviewed and assessed by two BCDs as high in quality and diagnostic of the underlying disease. Fifth, a third BCD reviewed the images and found no clear objections.

Although the gold-standard label for skin diseases such as cutaneous malignant neoplasm is histopathological diagnosis^96^, the majority of non-neoplastic skin diseases (including skin diseases) are considered readily diagnosable with an in-patient exam and a patient’s clinical history^97^. The images in this experiment come from external sources (textbooks, dermatology atlases, online search engines and dermatologist education materials) and were curated and confirmed to be correctly labeled by three BCDs, to the best of their knowledge, based on the visual features in the images.

As a post hoc quality review, three board-certified dermatologists reviewed the three most and least accurately diagnosed images for light and dark skin in each of the eight skin diseases. The analysis of these images by three BCDs indicates that the most accurately diagnosed images appear to be relatively classic presentations of each skin disease (for example a heliotrope sign and gottron papules for dermatomyositis, rashes of the hands and feet for secondary syphilis, bulls-eye rash for Lyme), while the least accurately diagnosed images appear to be atypical presentations.

As an additional quality-control measure, Extended Data Table 6 summarizes the sources from which we drew these images and how accurately BCDs identify the reference label across sources. Among the images of the main eight diseases that no BCD diagnosed correctly, 15% of those images come from dermatology textbooks. This is slightly higher than the proportion of textbook images in the 284 images of the eight diseases, which is 11%.

### Skin tone annotations

We annotated images by initially hiring crowdworkers to provide estimated FSTs for each image and then asking BCDs to update the FST label appropriately. The images are relatively balanced across FST, with 32% of images showing people with the two darkest FST labels (FST 5 and 6) and 68% showing people with the four lightest FST labels (FST 1–4). We define light and dark according to the original FST scale, which indicates FST 1–4 as ‘white’ and FST 5 and 6 as ‘black’ and ‘brown’^98^. Our findings are robust to comparisons between the three lightest and three darkest skin diseases, as well as comparisons between the two lightest and two darkest skin diseases. We note that the FST scale is imperfect (and its imperfections have been widely discussed^33,99–101^), but it remains a useful starting point for examining diagnostic accuracy disparities across skin tones.

### DLS development

To offer computer vision-based predictions of diagnoses, we trained a convolutional neural network to classify nine labels: the eight skin diseases of interest and another category. This neural network is a VGG-16 architecture pretrained on ImageNet, which is similar to the architecture used in ref. ^29^ and identical to the architecture of ref. ^31^. Following insights that fine-tuning on diverse data can close performance gaps between light and dark skin tones^32^, we fine-tuned the model on 31,219 diverse clinical dermatology images from the Fitzpatrick 17k dataset and an additional collection of images collected from textbooks, dermatology atlases and online search engines. The fine-tuning includes a number of transformations to images, including randomly resizing images to 256 × 256 pixels, randomly rotating images by 0–15°, randomly altering the brightness, contrast, saturation and hue of each image, randomly flipping the image horizontally or not, center cropping the image to 224 × 224 pixels, and normalizing the image arrays by the ImageNet means and standard deviations.

We evaluated the model on the 364 images in this experiment, which neither appear in the pre-training ImageNet data nor in the fine-tuning clinical dermatology images dataset, and we found the model to be 47% accurate at predicting the nine labels on the 364 images.

We did not compare the DLS system directly to physician performance, because the DLS system is trained to classify only nine labels, whereas physicians are tasked with diagnosing images without knowing the short list of what the possible skin diseases might be.

In this experiment, we refer to the VGG-16 architecture pretrained on ImageNet and fine-tuned on 31,219 clinical dermatology images as the ‘control DLS’.

In addition to the control DLS, we consider a ‘treatment DLS’, which is a Wizard of Oz classifier that is a synthetically enhanced version of the control DLS. To create the treatment DLS, we randomly re-assigned 65% of wrong classifications by the control DLS to be correct classifications, which resulted in a top-1 accuracy of 84%.

We note that the control and treatment DLSs are ‘fair’ classifiers from a disparate impact perspective. Both classifiers have relatively similar top-1 accuracies across skin tones on the eight diseases: the control DLS is 58% accurate on dark skin and 56% accurate on light skin on the eight main diseases, and the treatment DLS is 82% accurate on dark skin and 84% accurate on light skin on the eight main diseases.

Following the MI-CLAIM^102^ checklist, we examined the control DLS performance with two examination techniques. First, specialists examined the model’s performance across images and found that correct predictions often (but not always) correspond to classic presentations of a disease. Second, we examined the model’s performance across FST and we did not find meaningful differences in the model’s performance across skin types. In the context of the visual diagnosis of skin disease task, we did not find saliency maps particularly helpful for interpretability, because they highlighted skin lesions but did not provide any additional information on what differentiates one skin lesion from another.

### Randomization protocol

We randomly assigned the order in which images appear to participants for all images except the first. All participants see the same first image, and all subsequent images are drawn randomly from the remaining images.

We randomly assigned participants to two sets of control and treatment conditions. We randomly assigned participants to see suggestions from a control model (the 47% accurate model) or a synthetically enhanced treatment model (the 84% accurate model). We also randomly assigned the order in which the options appear for including or ignoring the suggestion in a participant’s differential diagnosis. The treatment group saw ‘Keep my differential’ on top and ‘Update my top prediction with [disease]’ on the bottom, as shown in Supplementary Fig. 4, whereas the control group saw the opposite, with ‘Update my top prediction with [disease]’ appearing on top. We randomly assigned participants to each condition with an equal weight. The number of BCDs and differential diagnoses for participants who completed the experiment for each condition (the control model and control interface, control model and treatment interface, treatment model and control interface, and treatment model and treatment interface) are 75 BCDs with 1,350 diagnoses, 64 BCDs with 1,150 diagnoses, 83 BCDs with 1,487 diagnoses, and 74 BCDs with 1,332 diagnoses, respectively. The number of PCPs and differential diagnoses for participants who completed the experiment for each condition (the control model and control interface, control model and treatment interface, treatment model and control interface, and treatment model and treatment interface) are 79 BCDs with 1,422 diagnoses, 85 BCDs with 1,530 diagnoses, 87 BCDs with 1,566 diagnoses, and 99 BCDs with 1,782 diagnoses, respectively.

### Participants

We recruited participants by word of mouth and by direct emails from Sermo, a secure digital (online) platform designed for physician networking and anonymous survey research, to their verified physician network. Sermo sent emails to 7,900 BCDs and 10,000 PCPs and offered US$10 for BCDs and US$5 for PCPs to complete the survey. In total, 68% of BCDs and 94% of PCPs in this experiment came from Sermo, and the rest came from authors reaching out to other physicians via email and social media. We recruited dermatology residents by identifying the email addresses of dermatology resident coordinators at 142 programs across the United States and requesting coordinators to forward an invitation to residents to participate in this study.

The countries with more than ten participants included the United States (551 total, with 167 BCDs, 47 dermatology residents, 295 PCPs and 42 other physicians), India (134 total, with 67 BCDs, 15 dermatology residents, 20 PCPs and 32 other physicians), Canada (91 total, with 18 BCDs, 1 dermatology resident, 59 PCPs and 13 other physicians), the United Kingdom (53 total, with 18 BCDs, 3 dermatology residents, 25 PCPs and 7 other physicians), Italy (45 total, with 13 BCDs, 18 dermatology residents, 6 PCPs and 8 other physicians), Germany (35 total, with 16 BCDs, 8 dermatology residents, 5 PCPs and 6 other physicians), Nigeria (30 total, with 3 dermatology residents, 6 PCPs and 21 other physicians), Brazil (22 total, with 11 BCDs, 4 dermatology residents, 5 PCPs and 2 other physicians), Spain (21 total, with 19 BCDs and 2 dermatology residents), Australia (18 total, with 3 BCDs, 1 dermatology resident, 8 PCPs and 6 other physicians), France (14 total, with 5 BCDs, 2 dermatology residents, 3 PCPs and 4 other physicians) and South Africa (14 total, with 3 BCDs, 7 PCPs and 4 other physicians).

In the pre-experiment survey, we asked physicians how many years they have practiced medicine, what is the distribution of their patients’ skin tone, what is the frequency of difficulty for diagnosing skin diseases in white and non-white patients, and how do they view the training they received for diagnosing skin diseases in patients with skin of color. In this experiment, 40% of physicians have been practicing medicine for 20 years or more, 26% have been practicing for 10 to 20 years, 22% have been practicing for 2 to 10 years, 3% have been practicing for 0 to 2 years, and the rest are doing residencies, fellowships or internships. In response to the question ‘How would you describe the distribution of your patients’ skin colors?’, 32% of participants responded about an equal portion of white and non-white patients, 43% responded mostly white patients, 2% responded all white patients, 15% responded mostly non-white, 7% responded all non-white patients, and 1% responded that the question is not applicable. This overall distribution is similar but slightly more diverse than the distribution for participants from the United States, which is skewed slightly more towards mostly white patients with 49% mostly white patients, 36% equal portion of white and non-white patients, and 13% mostly or all non-white patients.

We find PCPs report significantly higher rates of difficulty in diagnosing skin diseases for both light and dark skin than BCDs. Specifically, we find 8% of PCPs report difficulties diagnosing skin diseases in one in two white patients, and 15% of PCPs report difficulties diagnosing skin diseases in one in two non-white patients, whereas less than 3% of BCDs report difficulties in diagnosing skin diseases in one in two patients of any skin tone. For participants in the United States, 70% of BCDs and 72% of PCPs report the same diagnostic difficulty between white and non-white patients, and 10% of BCDs and 20% of PCPs report more difficulties in diagnosing non-white patients compared to white patients. When asked, ‘Do you feel you received sufficient training for diagnosing skin diseases in patients with skin of color (non-white patients)?’, 67% of all PCPs respond ‘no’ and 33% of all BCDs respond ‘no’ (similarly, 68% of US PCPs respond ‘no’ and 28% of US BCDs respond ‘no’).

### Annotating participants’ differential diagnoses

We collected 14,261 differential diagnoses, which include 2,348 unique text strings. As a function of our experimental interface, which asked participants to provide differential diagnoses in free-response text boxes supported by predictive text, 43% of the leading diagnosis text strings do not exactly match any of the text strings in the initial list of 445 diseases. However, the majority of these off-list responses are easily matched to the list. For example, 14% of the 14,261 leading diagnoses are ‘atopic dermatitis’, which we match to ‘atopic dermatitis (eczema)’ in the list, 4% of participants submitted ‘Lyme’, which we match to ‘lyme (erythema migrans)’ in the list, 3% of participants submitted ‘pityriasis rubra pilaris’, which we match to ‘pityriasis rubra pilaris (prp)’ in the list, and 3% of participants submitted ‘cutaneous t-cell lymphoma’, which we match to ‘cutaneous t-cell lymphoma (ctcl)’ in the list). The remaining 19% of leading diagnoses match 1,447 unique text strings. To evaluate diagnostic accuracy as accurately as possible, we reviewed all diagnoses and marked responses as correct if they appear to be misspellings or shorthand for the correct answer. For example, we included the following answers as correct for lichen planus: lichen planus, lichen ruber planus, lichens planus, lichen plan, lichen planes, lichen planhs, lichen planis, lichen plannus, lichen plans, lichen planus linearis, lichen planus, luchen planus, lichen planus, lichen plane, linear lichen planus, linen planu and liquen plano. As a second example, we included the following answers as correct for CTCL: cutaneous t-cell lymphoma, t cell lymphoma, cutaneous t cell lymphoma, cutaneous t cell, ctcl, mycosis fungoides, lymphoma, mucositá fungoide, micosi fungoide, myocses fungoides, mycosis fungiodies, mycoses fungoides, plaque type, mf, cuttaneoua t-cell lymph, linfoma, linfoma células t, linfoma t, lmphoma, lymphome, malignant skin cancer, t cell lyphoma, t-cell lyphoma, mucosis fungoides, mycoses fungoides, mycoses glfungoide, mycosis, mycosis fongicide, mycosis fungoides/ctcl, mycosis fungoidis, mycosis fungoidus, micose fungoide, micosis fungoide, micosis fungoides, cutaneous t-cell lymphoma (ctcl), ctcl (cutaneous t-cell lymphoma), cutaneous t-cell lymphoma, t-cell lymphoma, cutaneous lymphoma and cutaneous lympoma.

### Gamification designs

We designed the experiment with gamification ingredients as have been articulated in ref. ^103^, such as feedback, rewards, competition and clear rules. In particular, we provided feedback after every response on the reference label as well as the most common incorrect answer by other participants. When participants’ differential diagnosis included the reference label, we displayed a brief digital fireworks show on the screen. We informed participants that we would show them how they compare against the DLS system and other physicians after they provided ten differential diagnoses. The majority of participants completed at least one additional differential diagnosis after completing ten diagnoses and seeing how their performance compared to others. In Supplementary Fig. 8, we present the rate at which each group of participants continued participating in the experiment after completing their first ten responses. After 20 responses, over 10% of participants in each physician category were participating.

### Standards for Reporting Diagnostic Accuracy Studies

The updated Standards for Reporting Diagnostic Accuracy Studies (STARD) 2015 guidelines are designed to help readers of diagnostic accuracy studies recognize for which patient groups and settings a diagnostic accuracy study is relevant^104,105^. Although this study focuses on physician diagnostic accuracy, which differs substantially from standard diagnostic accuracy studies that focus on medical test accuracy, we followed the STARD 2015 checklist to clarify the study objectives, experimental design, analysis, limitations and implications for clinical dermatology practice and designing physician–machine partnerships.

### Software and code

We hosted the store-and-forward digital experiment at https://diagnosing -diagnosis.media.mit.edu using a custom website built in Python using the Flask web framework. All experimental data are collected based on how participants interact with the website.

The data analysis was performed in Python 3.9.6 with the libraries pandas 1.4.0, matplotlib 3.2.2, seaborn 0.11.1, numpy 1.18.5, scipy 1.5.0, statsmodels, stargazer 0. 11.1 and sklearn 0.0.5.

The DLS was trained using PyTorch, and additional details are presented in the DLS development subsection in the Methods.

### Reporting summary

Further information on research design is available in the Nature Portfolio Reporting Summary linked to this Article.

## Online content

Any methods, additional references, Nature Portfolio reporting summaries, source data, extended data, supplementary information, acknowledgements, peer review information; details of author contributions and competing interests; and statements of data and code availability are available at 10.1038/s41591-023-02728-3.

### Supplementary information

Supplementary Information Supplementary Tables 1–4 and Figs. 1–8.

Reporting Summary

## Figures and Tables

**Extended Data Table 1:**
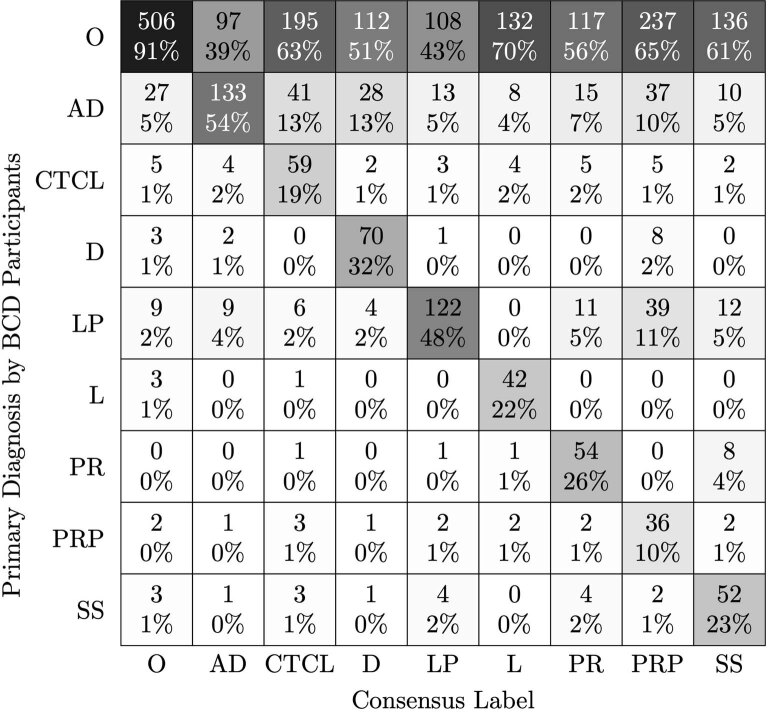
Confusion Matrix for BCDs

Confusion matrix comparing consensus labels verified by three board-certified dermatologists with the primary diagnoses submitted by board-certified dermatologists. The labels in the confusion matrix refer to the following: O is other and includes 38 skin diseases, AD is atopic dermatitis, CTCL is cutaneous t-cell lymphoma, D is dermatomyositis, LP is lichen planus, L is Lyme, PR is pityriasis rosea, PRP is pityriasis rubra pilaris, and SS is secondary syphilis.

**Extended Data Table 2:**
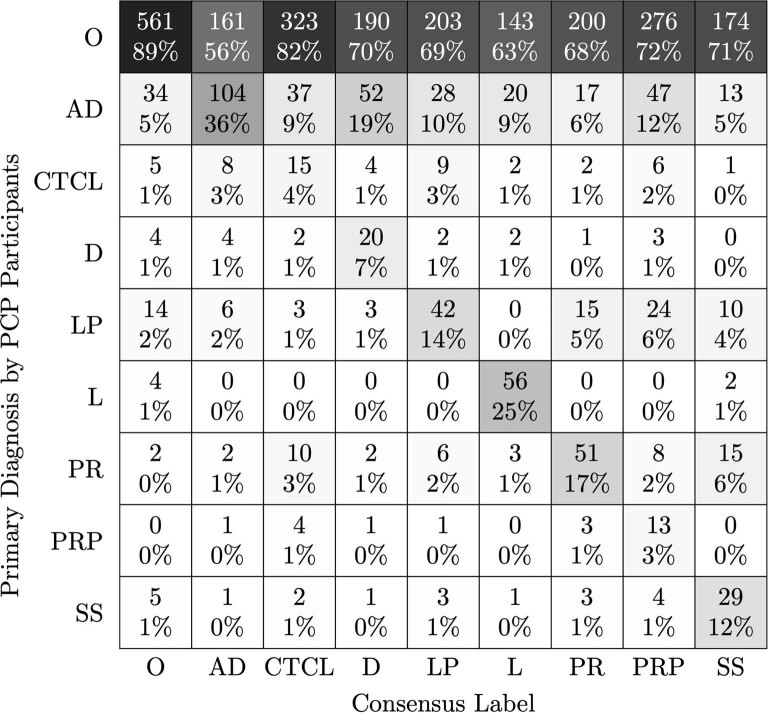
Confusion Matrix for PCPs

Confusion matrix comparing consensus labels verified by three board-certified dermatologists with the primary diagnoses submitted by primary-care physicians. The labels in the confusion matrix refer to the following: O is other and includes 38 skin diseases, AD is atopic dermatitis, CTCL is cutaneous t-cell lymphoma, D is dermatomyositis, LP is lichen planus, L is Lyme, PR is pityriasis rosea, PRP is pityriasis rubra pilaris, and SS is secondary syphilis.

**Extended Data Table 3:**
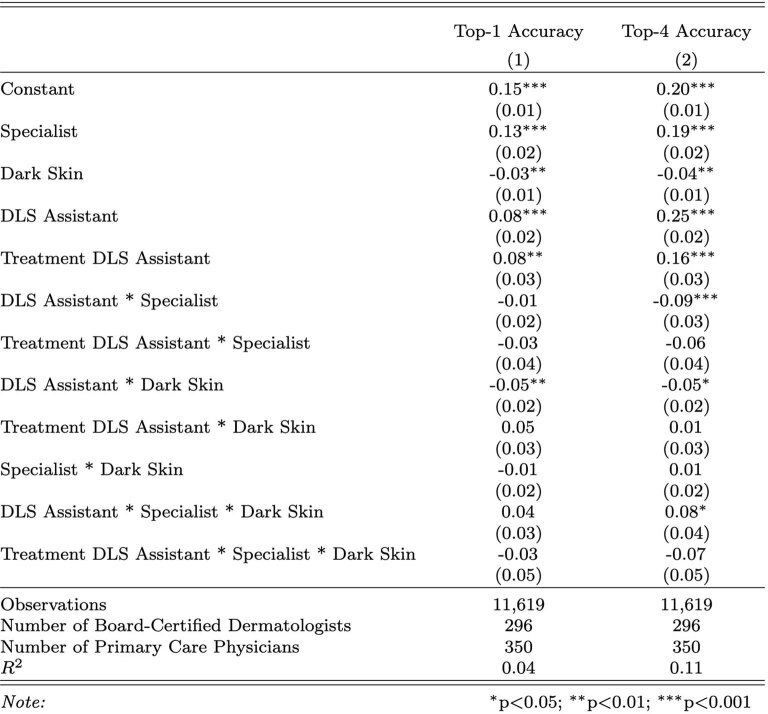
Regression of Physician Expertise Level, Patient Skin Color, and DLS Assistance on Diagnostic Accuracy

Ordinary Least Squares regressions with robust standard errors clustered on physician participants. This regression includes only board-certified dermatologist (BCD) and primary-care physician (PCP) participants. We code the Specialist, Dark Skin, AI Assistance binary variables as follows: Specialist equals 1 for BCDs and 0 for PCPs, Dark Skin equals 1 for FST 5 and 6 and 0 for FST 1 to 4, and AI Assistance equals 1 for participant responses with access to the DLS prediction and 0 for participant responses before access to the DLS predictions. The coefficients represent the change in the dependent variable for a one-unit change in the independent variable while holding everything else constant. The numbers in parentheses are standard errors indicating the variability of coefficient estimates. *, **, and *** indicates the p-value from the ordinary least-squares regression is less than 0.05, 0.01, and 0.001.

**Extended Data Table 4:**
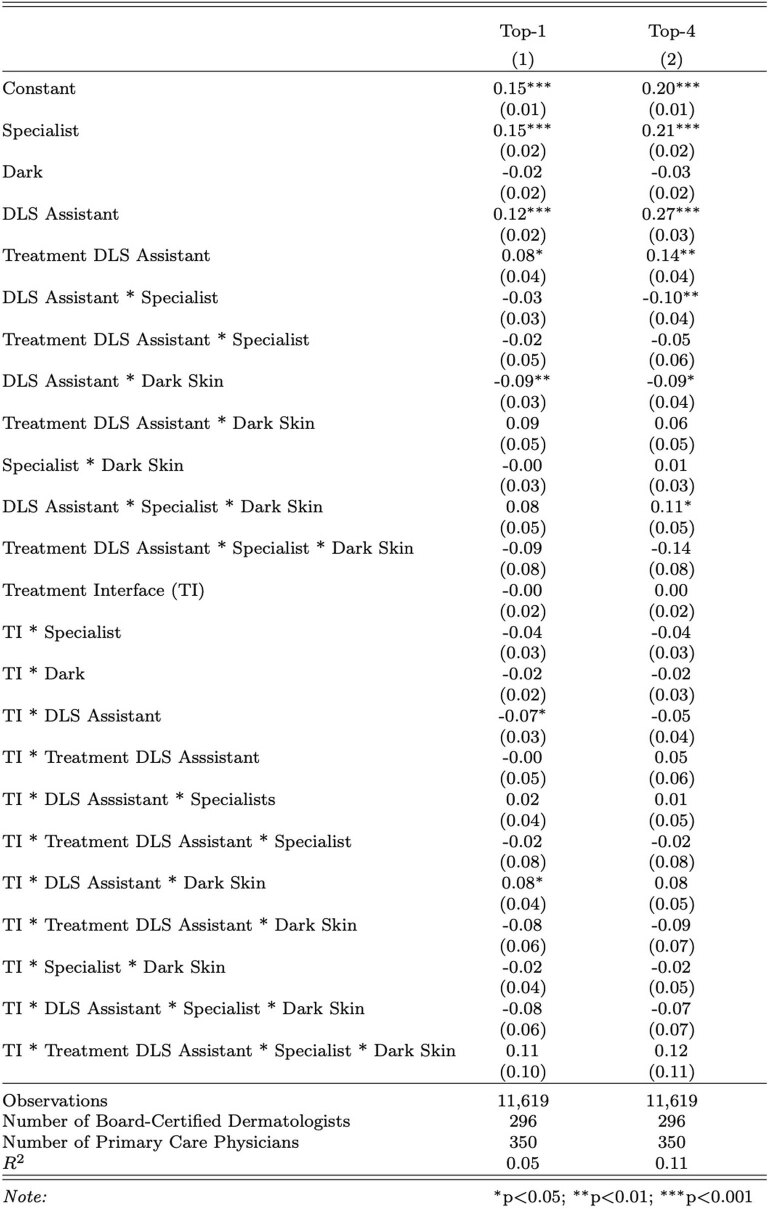
Regression of Physician Expertise Level, Patient Skin Color, and DLS Assistance with User Interface Interactions on Diagnostic Accuracy

Ordinary Least Squares regressions with robust standard errors clustered on physician participants. This regression includes only board-certified dermatologist (BCD) and primary-care physician (PCP) participants. We code the Specialist, Dark Skin, AI Assistance binary variables as follows: Specialist equals 1 for BCDs and 0 for PCPs, Dark Skin equals 1 for FST 5 and 6 and 0 for FST 1 to 4, AI Assistance equals 1 for participant responses with access to the DLS prediction and 0 for participant responses before access to the DLS predictions, and Treatment Interface (TI) equals 1 for interface order with “Keep my differential” on Top and 0 for the opposite interface order. The coefficients represent the change in the dependent variable for a one-unit change in the independent variable while holding everything else constant. The numbers in parentheses are standard errors indicating the variability of coefficient estimates. *, **, and *** indicates the p-value from the ordinary least-squares regression is less than 0.05, 0.01, and 0.001.

**Extended Data Table 5:**
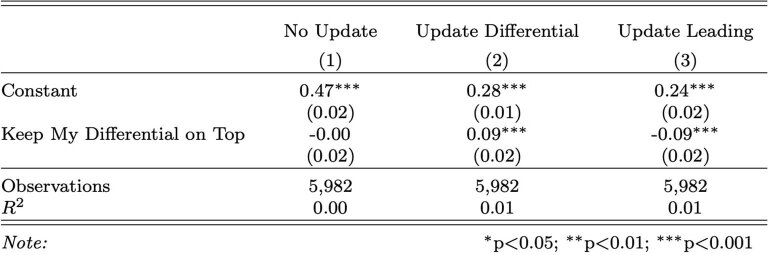
Average Treatment Effects of Reversed Order of Decision Support Buttons

Average treatment effects of user interaction design with “Keep My Differential” on top (as opposed to on bottom) based on ordinary least-squares regressions with robust standard errors clustered on participants. The coefficients represent the change in the dependent variable for a one-unit change in the independent variable while holding everything else constant. The numbers in parentheses are standard errors indicating the variability of coefficient estimates. *, **, and *** indicates the p-value from the ordinary least-squares regression is less than 0.05, 0.01, and 0.001.

**Extended Data Table 6:**
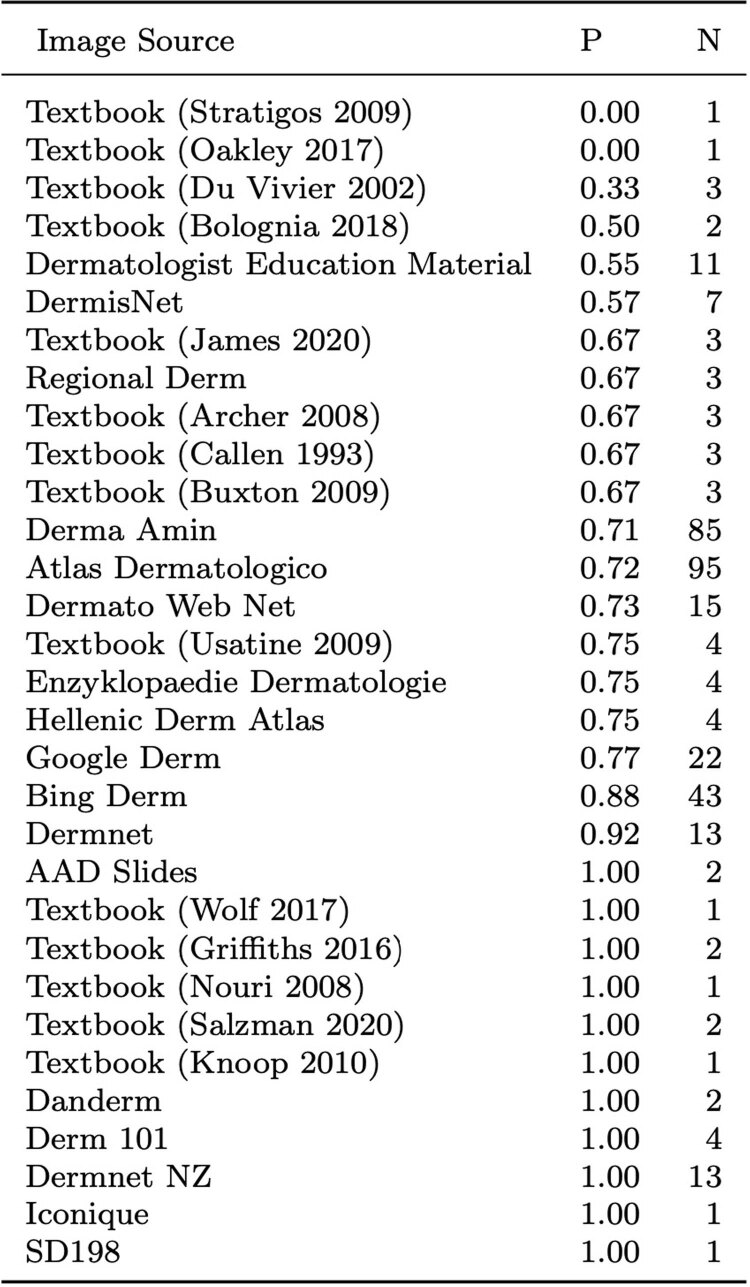
Image Sources

Table of image sources with P indicating the proportion of images from a particular source in which at least one board-certified dermatologist provided a top-3 diagnosis matching the source image′s label. N indicates the number of images from each source.

## Data Availability

The experimental data necessary to reproduce the results of this study are available on ResearchBox at https://researchbox.org/1802. The 364 images used in the experiment are available at 10.5281/zenodo.10070478 to registered Zenodo users who agree to only use this dataset for scientific and medical purposes and delete the data from their device once their research is complete.

## Code availability

The code to reproduce the results of this study is available on ResearchBox at https://researchbox.org/1802.

## References

[1] Jain A (2021). Development and assessment of an artificial intelligence-based tool for skin condition diagnosis by primary care physicians and nurse practitioners in teledermatology practices. JAMA Netw. Open.

[2] Tschandl P (2020). Human–computer collaboration for skin cancer recognition. Nat. Med..

[3] Wiens J (2019). Do no harm: a roadmap for responsible machine learning for health care. Nat. Med..

[4] Varoquaux G, Cheplygina V (2022). Machine learning for medical imaging: methodological failures and recommendations for the future. NPJ Digit. Med..

[5] Patel BN (2019). Human–machine partnership with artificial intelligence for chest radiograph diagnosis. NPJ Digit. Med..

[6] Kostick-Quenet KM, Gerke S (2022). AI in the hands of imperfect users. NPJ Digit. Med..

[7] Chen H, Gomez C, Huang C-M, Unberath M (2022). Explainable medical imaging AI needs human-centered design: guidelines and evidence from a systematic review. NPJ Digit. Med..

[8] Marchetti MA (2023). Prospective validation of dermoscopy-based open-source artificial intelligence for melanoma diagnosis (PROVE-AI study). NPJ Digit. Med..

[9] Dvijotham K (2023). Enhancing the reliability and accuracy of AI-enabled diagnosis via complementarity-driven deferral to clinicians. Nat. Med.

[10] Campero, A. et al. A test for evaluating performance in human-computer systems. Preprint at https://arxiv.org/abs/2206.12390 (2022).

[11] Lebovitz S, Lifshitz-Assaf H, Levina N (2022). To engage or not to engage with AI for critical judgments: how professionals deal with opacity when using AI for medical diagnosis. Organ. Sci..

[12] Gaube S (2021). Do as AI say: susceptibility in deployment of clinical decision-aids. NPJ Digit. Med..

[13] Seyyed-Kalantari L, Zhang H, McDermott MBA, Chen IY, Ghassemi M (2021). Underdiagnosis bias of artificial intelligence algorithms applied to chest radiographs in under-served patient populations. Nat. Med..

[14] Groh M, Epstein Z, Firestone C, Picard R (2022). Deepfake detection by human crowds, machines and machine-informed crowds. Proc. Natl Acad. Sci. USA.

[15] DeCamp M, Lindvall C (2023). Mitigating bias in AI at the point of care. Science.

[16] Rajpurkar P, Chen E, Banerjee O, Topol EJ (2022). AI in health and medicine. Nat. Med..

[17] Williams DR, Wyatt R (2015). Racial bias in health care and health: challenges and opportunities. JAMA.

[18] Dehon E (2017). A systematic review of the impact of physician implicit racial bias on clinical decision making. Acad. Emerg. Med..

[19] Obermeyer Z, Powers B, Vogeli C, Mullainathan S (2019). Dissecting racial bias in an algorithm used to manage the health of populations. Science.

[20] Singh, M. & Venkataramani, A. *Capacity Strain and Racial Disparities in Hospital Mortality*. Technical Report (National Bureau of Economic Research, 2022).

[21] Zou J, Gichoya JW, Ho DE, Obermeyer Z (2023). Implications of predicting race variables from medical images. Science.

[22] Alvarado SM, Feng H (2020). Representation of dark skin images of common dermatologic conditions in educational resources: a cross-sectional analysis. J. Am. Acad. Dermatol..

[23] Adelekun A, Onyekaba G, Lipoff JB (2020). Skin color in dermatology textbooks: an updated evaluation and analysis. J. Am. Acad. Dermatol..

[24] Lester J, Shinkai K (2019). Diversity and inclusivity are essential to the future of dermatology. Cutis.

[25] Lester JC, Jia JL, Zhang L, Okoye GA, Linos E (2020). Absence of images of skin of colour in publications of COVID-19 skin manifestations. Br. J. Dermatol..

[26] Fenton A (2020). Medical students’ ability to diagnose common dermatologic conditions in skin of color. J. Am. Acad. Dermatol..

[27] Diao JA, Adamson AS (2022). Representation and misdiagnosis of dark skin in a large-scale visual diagnostic challenge. J. Am. Acad. Dermatol..

[28] Daneshjou R, Smith MP, Sun MD, Rotemberg V, Zou J (2021). Lack of transparency and potential bias in artificial intelligence data sets and algorithms: a scoping review. JAMA Dermatol..

[29] Esteva A (2017). Dermatologist-level classification of skin cancer with deep neural networks. Nature.

[30] Liu Y (2020). A deep learning system for differential diagnosis of skin diseases. Nat. Med..

[31] Groh, M. et al. Evaluating deep neural networks trained on clinical images in dermatology with the Fitzpatrick 17k dataset. In *Proc. 2021 IEEE*/*CVF Conference on Computer Vision and Pattern Recognition Workshops* (*CVPRW*) 1820–1828 (IEEE, 2021).

[32] Daneshjou R (2022). Disparities in dermatology AI performance on a diverse, curated clinical image set. Sci. Adv..

[33] Groh M, Harris C, Daneshjou R, Badri O, Koochek A (2022). Towards transparency in dermatology image datasets with skin tone annotations by experts, crowds and an algorithm. Proc. ACM Hum. Comput. Interact..

[34] Sagers, L. W. et al. Improving dermatology classifiers across populations using images generated by large diffusion models. In *NeurIPS 2022 Workshop on Synthetic Data for Empowering ML Research* (2022); https://openreview.net/forum?id=Vzdbjtz6Tys

[35] Chen IY, Szolovits P, Ghassemi M (2019). Can AI help reduce disparities in general medical and mental health care?. AMA J. Ethics.

[36] Rand DG (2012). The promise of mechanical turk: how online labor markets can help theorists run behavioral experiments. J. Theor. Biol..

[37] Paolacci G, Chandler J, Ipeirotis PG (2010). Running experiments on Amazon mechanical turk. Judgm. Decis. Mak..

[38] Murali Doraiswamy P, Blease C, Bodner K (2020). Artificial intelligence and the future of psychiatry: insights from a global physician survey. Artif. Intell. Med..

[39] Long B, Simson J, Bux´o-Lugo A, Watson DG, Mehr SA (2023). How games can make behavioural science better. Nature.

[40] Almaatouq, A. et al. Beyond playing 20 questions with nature: integrative experiment design in the social and behavioral sciences. *Behav. Brain Sci.*10.1017/S0140525X22002874 (2022). 10.1017/S0140525X22002874 36539303

[41] Liu X (2022). The medical algorithmic audit. Lancet Digit. Health.

[42] Buolamwini, J. & Gebru, T. Gender shades: intersectional accuracy disparities in commercial gender classification. In *Proc. 1st Conference on Fairness*, *Accountability and Transparency* Vol. 81, 77–91 (PMLR, 2018).

[43] Mullainathan S, Obermeyer Z (2022). Diagnosing physician error: a machine learning approach to low-value health care. Q. J. Econ..

[44] Schiff GD (2009). Diagnostic error in medicine: analysis of 583 physician-reported errors. Arch. Intern. Med..

[45] Nolen LS (2020). How medical education is missing the bull’s-eye. N. Engl. J. Med..

[46] Fix AD, Peña CA, Strickland GT (2000). Racial differences in reported Lyme disease incidence. Am. J. Epidemiol..

[47] Croce EA, Levy ML, Adamson AS, Matsui EC (2021). Reframing racial and ethnic disparities in atopic dermatitis in Black and Latinx populations. J. Allergy Clin. Immunol..

[48] Federman DG, Kirsner RS (1997). The abilities of primary care physicians in dermatology: implications for quality of care. Am. J. Manag. Care.

[49] Federman DG, Concato J, Kirsner RS (1999). Comparison of dermatologic diagnoses by primary care practitioners and dermatologists: a review of the literature. Arch. Fam. Med..

[50] Tran H, Chen K, Lim AC, Jabbour J, Shumack S (2005). Assessing diagnostic skill in dermatology: a comparison between general practitioners and dermatologists. Australas. J. Dermatol..

[51] Chen SC (2006). Diagnosing and managing cutaneous pigmented lesions: primary care physicians versus dermatologists. J. Gen. Intern. Med..

[52] Moreno G, Tran H, Chia ALK, Lim A, Shumack S (2007). Prospective study to assess general practitioners’ dermatological diagnostic skills in a referral setting. Australas. J. Dermatol..

[53] Snoswell C, Finnane A, Janda M, Soyer HP, Whitty JA (2016). Cost-effectiveness of store-and-forward teledermatology: a systematic review. JAMA Dermatol..

[54] Lebovitz S, Levina N, Lifshitz-Assaf H (2021). Is AI ground truth really `true'? The dangers of training and evaluating AI tools based on experts' know-what. Manag. Inf. Syst. Q.

[55] Schiff GD (2018). Ten principles for more conservative, care-full diagnosis. Ann. Intern. Med..

[56] Widner K (2023). Lessons learned from translating AI from development to deployment in healthcare. Nat. Med..

[57] Muse ED (2018). From second to hundredth opinion in medicine: a global consultation platform for physicians. NPJ Digit. Med..

[58] Centola D, Guilbeault D, Sarkar U, Khoong E, Zhang J (2021). The reduction of race and gender bias in clinical treatment recommendations using clinician peer networks in an experimental setting. Nat. Commun..

[59] Centola D (2023). Experimental evidence for structured information-sharing networks reducing medical errors. Proc. Natl Acad. Sci. USA.

[60] Hasan, E., Eichbaum, Q., Seegmiller, A. C., Stratton, C. & Trueblood, J. S. Harnessing the wisdom of the confident crowd in medical image decision-making. *Decision*10.1037/dec0000210 (2023).

[61] The All of Us Research Program Investigators. (2019). The ‘all of us’ research program. N. Engl. J. Med..

[62] Sacotte R, Silverberg JI (2018). Epidemiology of adult atopic dermatitis. Clin. Dermatol..

[63] Maghfour J (2022). Demographic patterns and increasing incidence of cutaneous T-cell lymphoma in Louisiana. JAMA Oncol..

[64] Bolender CM (2022). Incidence of dermatomyositis in a nationwide cohort study of US veterans. JAMA Dermatol..

[65] Li C (2020). Global prevalence and incidence estimates of oral lichen planus: a systematic review and meta-analysis. JAMA Dermatol..

[66] Nelson CA (2015). Incidence of clinician-diagnosed Lyme disease, United States, 2005-2010. Emerg. Infect. Dis..

[67] Joshi TP, Calderara GA, Lipoff JB (2022). Prevalence of pityriasis rosea in the United States: a cross-sectional study using the all of us database. JAAD Int..

[68] Schmidt R, Carson PJ, Jansen RJ (2019). Resurgence of syphilis in the United States: an assessment of contributing factors. Infect. Dis. Res. Treat..

[69] Ross NA (2016). Epidemiologic, clinicopathologic, diagnostic, and management challenges of pityriasis rubra pilaris: a case series of 100 patients. JAMA Dermatol..

[70] Freire da Silva, S. Atlas dermatologico; http://atlasdermatologico.com.br/

[71] AlKattash, J. A. Dermaamin; https://www.dermaamin.com/site/

[72] Bolognia, J. L., Schaffer, J. V. & Cerroni, L. *Dermatología* (Elsevier, 2018).

[73] Griffiths, C., Barker, J., Bleiker, T. O., Chalmers, R. & Creamer, D. *Rook’s Textbook of Dermatology* (Wiley, 2016).

[74] Du Vivier, A. *Atlas of Clinical Dermatology* (Elsevier, 2002).

[75] Archer, C. B. *Ethnic Dermatology*: *Clinical Problems and Skin Pigmentation* (CRC Press, 2008).

[76] Nouri, K. et al. In *Skin Cancer* 61–81 (McGraw Hill Medical, 2008).

[77] Salzman H (2020). The color atlas and synopsis of family medicine. Fam. Med..

[78] Knoop, K. J. et al. (eds) *The Atlas of Emergency Medicine* (McGraw Hill, 2010).

[79] Usatine, R. P., Smith, M. A., Mayeaux, E. J. & Chumley, H. S. *The Color Atlas of Family Medicine* (McGraw Hill Education/Medical, 2013).

[80] Buxton, P. K. & Morris-Jones, R. In *ABC of Dermatology* 24–35 (Wiley Blackwell, 2009).

[81] Callen, J. P., Greer, K. E., Hood, A. F., Paller, A. S. & Swinyer, L. J. *Color Atlas of Dermatology* (Saunders, 1993).

[82] Kane, K. S., Lio, P. A. & Stratigos, A. *Color Atlas and Synopsis of Pediatric Dermatology* (McGraw Hill Education/Medical, 2009).

[83] Oakley, A. *Dermatology Made Easy* (Scion, 2017).

[84] Anon. DermIS, dermis.net; https://www.dermis.net/dermisroot/en/home/index.htm (accessed 17 February 2023).

[85] Arnold, H. L., Odom, R. B., Andrews, G. C. & James, W. D. *Andrews’ Diseases of the Skin*: *Clinical Dermatology* (Saunders, 1990).

[86] Anon. Regionalderm.com; https://www.regionalderm.com/contact.info.html (accessed 17 February 2023).

[87] Anon. Altmeyers Enzyklopädie – Fachbereich Dermatologie; https://www.altmeyers.org/de/dermatologie (accessed 17 February 2023).

[88] Anon. Hellenic Dermatological Atlas; http://www.hellenicdermatlas.com/en/ (accessed 17 February 2023).

[89] We are currently Redesigning Dermnet Skin Disease Atlas – dermnet.com; https://dermnet.com/ (accessed 17 February 2023).

[90] Anon. Atlas of Dermatology; https://www.kkh.dk/atlas/index.html (accessed 17 February 2023).

[91] Anon. Derm101; https://www.emailmeform.com/builder/form/Ne0j8da9bb7U4h6t1f (accessed 17 February 2023).

[92] Anon. DermWeb; http://www.dermweb.com/photo atlas/ (accessed 17 February 2023).

[93] Sun, X., Yang, J., Sun, M. & Wang, K. A benchmark for automatic visual classification of clinical skin disease images. In *Proc. Computer Vision ECCV 2016*: *14th European Conference Part VI 14* 206–222 (Springer, 2016).

[94] Anon. Iconotheque numerique de l’universite libre de Bruxelles; https://icono.ulb.ac.be/ (accessed 17 February 2023).

[95] Kilikita, J. Rosacea is common in dark skin, too. here’s what you need to know. https://www.refinery29.com/en-gb/rosacea-dark-skin

[96] Daneshjou R, He B, Ouyang D, Zou JY (2021). How to evaluate deep learning for cancer diagnostics—factors and recommendations. Biochim. Biophys. Acta.

[97] Harvey NT, Chan J, Wood BA (2017). Skin biopsy in the diagnosis of inflammatory skin disease. Aust. Fam. Physician.

[98] Fitzpatrick TB (1988). The validity and practicality of sun-reactive skin types I through VI. Arch. Dermatol..

[99] Ware OR, Dawson JE, Shinohara MM, Taylor SC (2020). Racial limitations of Fitzpatrick skin type. Cutis.

[100] Okoji UK, Taylor SC, Lipoff JB (2021). Equity in skin typing: why it is time to replace the Fitzpatrick scale. Br. J. Dermatol..

[101] Monk EP (2015). The cost of color: skin color, discrimination and health among African-Americans. Am. J. Sociol..

[102] Norgeot B (2020). Minimum information about clinical artificial intelligence modeling: the MI-CLAIM checklist. Nat. Med..

[103] Reeves, B. & Read, J. L. *Total Engagement*: *How Games and Virtual Worlds are Changing the Way People Work and Businesses Compete* (Harvard Business Press, 2009).

[104] Bossuyt PM (2015). STARD 2015: an updated list of essential items for reporting diagnostic accuracy studies. Clin. Chem..

[105] Cohen JF (2016). STARD 2015 guidelines for reporting diagnostic accuracy studies: explanation and elaboration. BMJ Open.

