# Supplementary material for: Deep learning-aided decision support for diagnosis of skin disease across skin tones
Source: Nat Med. 2024 Feb 5;30(2):573–83. doi: 10.1038/s41591-023-02728-3 (PMC10878981; doi:10.1038/s41591-023-02728-3)
Supplement: Supplementary file 1 — Supplementary Tables 1–4 and Figs. 1–8. [file 41591_2023_2728_MOESM1_ESM.pdf]

nature medicine

Article

https://doi.org/10.1038/s41591-023-02728-3

Deep learning-aided decision support for diagnosis of skin disease across skin tones

In the format provided by the authors and unedited

Supplemental Information

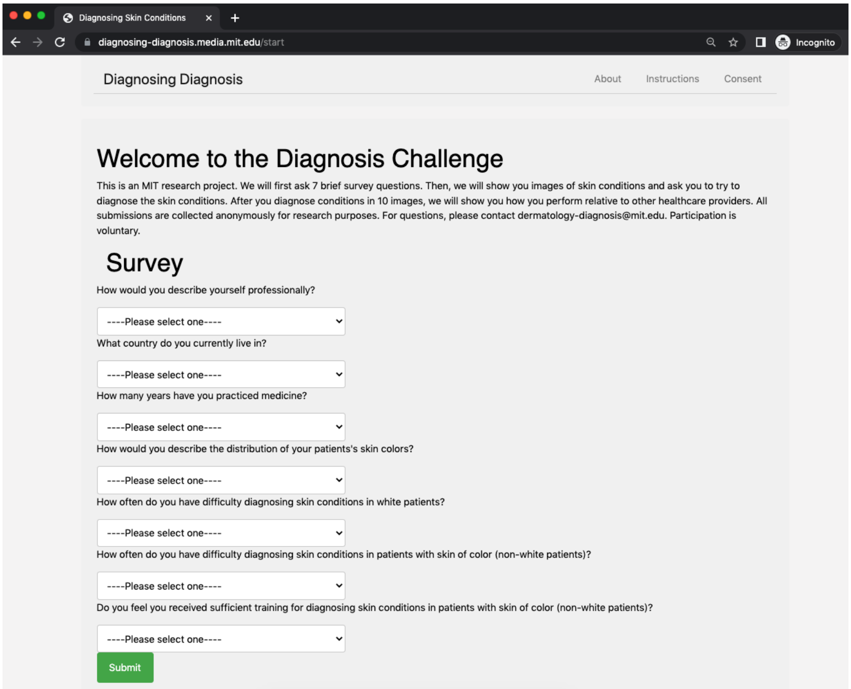

Supplementary Information Figure 1: Welcome Page. Screenshot from the Diagnosing Diagnosis experiment website showing the welcome landing page

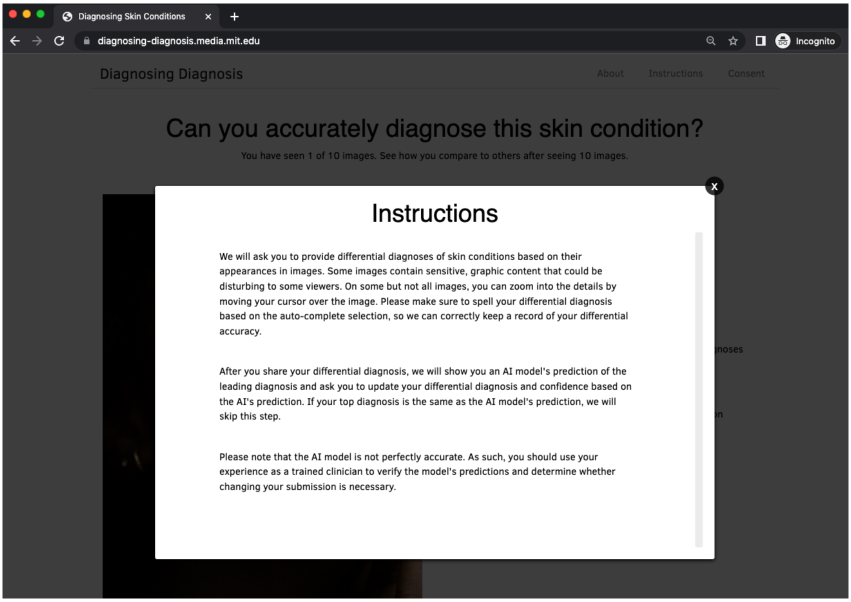

Supplementary Information Figure 2: Instructions Page. Screenshot from the Diagnosing Diagnosis experiment website showing the instructions.

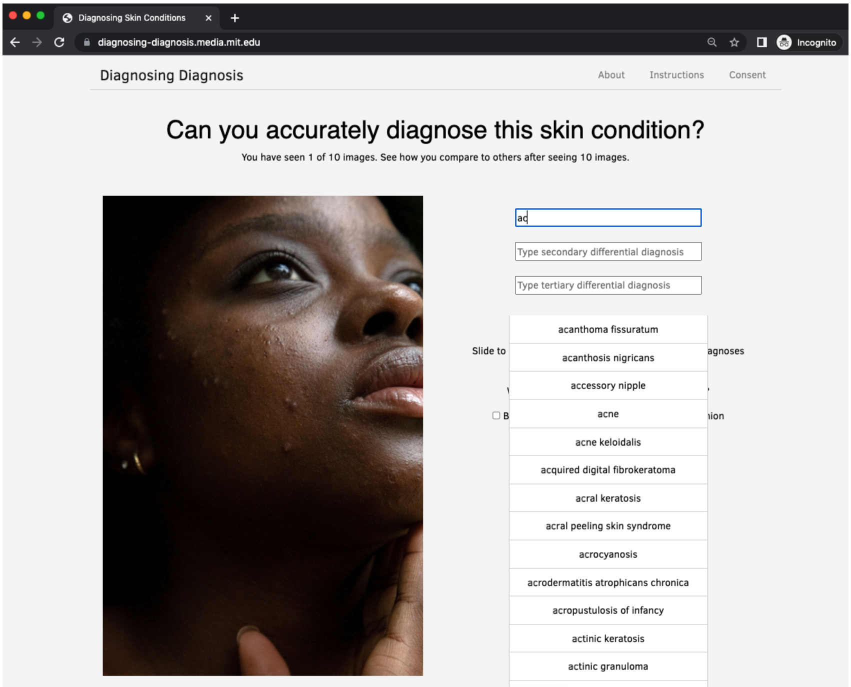

Supplementary Information Figure 3: Differential Diagnosis Page with Predictive Text. Screenshot from the Diagnosing Diagnosis experiment website showing predictive text for selecting diagnoses.

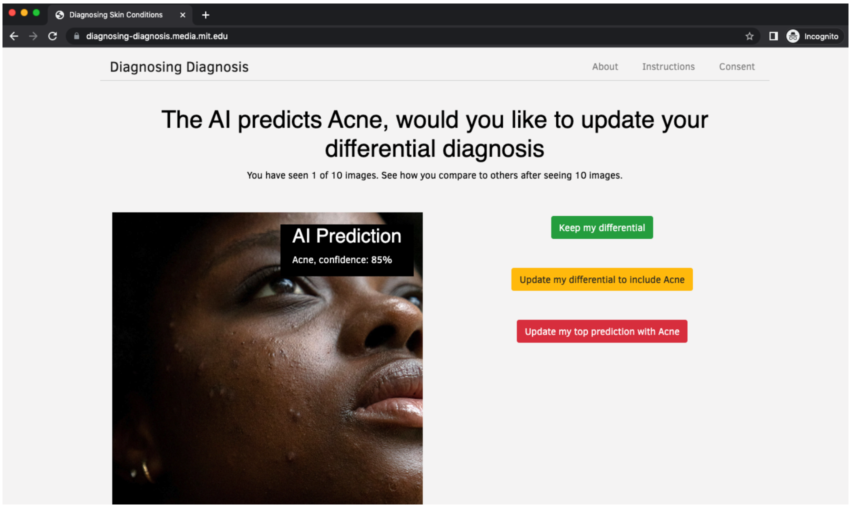

Supplementary Information Figure 4: Decision Support Page. Screenshot from the Diagnosing Diagnosis experiment website showing the DLS suggestion. Participants are randomly assigned to either see the three options in the order presented or the reverse order with "Update my top prediction..." on top, "Update my differential" in the middle, and "Keep my differential" on the bottom.

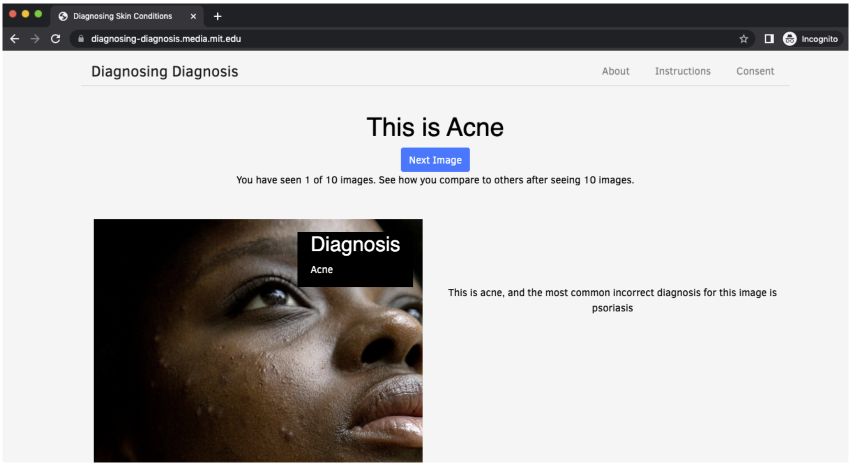

Supplementary Information Figure 5: Feedback Page. Screenshot from the Diagnosing Diagnosis experiment website showing the feedback based on the original label.

	Dependent variable: BCD Top-1 Accuracy			
	R1	R2	R3	R4
	(1)	(2)	(3)	(4)
Constant	0.29***	0.32***	0.28***	0.29***
	(0.01)	(0.02)	(0.01)	(0.01)
Fitzpatrick Skin Type 5 and 6	-0.05*	-0.06*	-0.04*	-0.05***
	(0.02)	(0.03)	(0.02)	(0.01)
Observations	2,660	1,215	2,855	4,976
Number of Dermatologists	296	135	374	377
Number of Images	363	363	363	363
R^2	0.00	0.00	0.00	0.00
Note:		*p<0.05; **p<0.01; ***p<0.001		

Supplementary Information Table 1: Robustness Check for Regression of Patients' Fitzpatrick Skin Type on BCD Physicians' Top-1 Accuracy. Robustness check for top-1 accuracy disparities across skin tone based on alternative inclusion criteria for board-certified dermatologists. This table shows ordinary least squares regressions with robust standard errors clustered on physician participants. The dependent variable is top-1 accuracy. Column (1) includes the first 10 differential diagnoses of images by participants who passed the attention check and completed at least 10 differential diagnoses of images (2) includes the subset of participants from (1) who live in the United States (3) includes the subset of participants from (1) and also includes participants who provided fewer than 10 differential diagnoses of images, and (4) includes all responses of all physician participants who pass the attention check. The coefficients represent the change in the dependent variable for a one-unit change in the independent variable while holding everything else constant. The numbers in parentheses are standard errors indicating the variability of coefficient estimates. *, **, and *** indicates the p-value from the ordinary least squares regression is less than 0.05, 0.01, and 0.001.

	Dependent variable: BCD Top-3 Accuracy			
	R1	R2	R3	R4
	(1)	(2)	(3)	(4)
Constant	0.40***	0.43***	0.38***	0.38***
	(0.01)	(0.02)	(0.01)	(0.02)
Fitzpatrick Skin Type 5 and 6	-0.03	-0.03	-0.03	-0.03*
	(0.02)	(0.03)	(0.02)	(0.01)
Observations	2,660	1,215	2,855	4,976
Number of Dermatologists	296	135	374	377
Number of Images	363	363	363	363
R^2	0.00	0.00	0.00	0.00
Note:		*p<0.05; **p<0.01; ***p<0.001		

Supplementary Information Table 2: Robustness Check for Regression of Patients' Fitzpatrick Skin Type on BCD Physicians' Top-3 Accuracy. Robustness check for top-3 accuracy disparities across skin tone based on alternative inclusion criteria for board-certified dermatologists. This table shows ordinary least squares regressions with robust standard errors clustered on physician participants. The dependent variable is top-1 accuracy. Column (1) includes the first 10 differential diagnoses of images by participants who passed the attention check and completed at least 10 differential diagnoses of images (2) includes the subset of participants from (1) who live in the United States (3) includes the subset of participants from (1) and also includes participants who provided fewer than 10 differential diagnoses of images, and (4) includes all responses of all physician participants who pass the attention check. The coefficients represent the change in the dependent variable for a one-unit change in the independent variable while holding everything else constant. The numbers in parentheses are standard errors indicating the variability of coefficient estimates. *, **, and *** indicates the p-value from the ordinary least squares regression is less than 0.05, 0.01, and 0.001.

	Dependent variable: PCP Top-1 Accuracy			
	(1)	(2)	(3)	(4)
Constant	0.15***	0.16***	0.16***	0.17***
	(0.01)	(0.01)	(0.01)	(0.01)
Fitzpatrick Skin Type 5 and 6	-0.03**	-0.05**	-0.03**	-0.04***
	(0.01)	(0.02)	(0.01)	(0.01)
Observations	3,150	2,052	3,352	4,999
Number of Primary Care Providers	350	228	434	441
Number of Images	363	363	363	363
R^2	0.00	0.00	0.00	0.00
Note:		*p<0.05; **p<0.01; ***p<0.001		

Supplementary Information Table 3: Robustness Check for Regression of Patients' Fitzpatrick Skin Type on PCP Physicians' Top-1 Accuracy. Robustness check for top-1 accuracy disparities across skin tone based on alternative inclusion criteria for primary care physicians. This table shows ordinary least squares regressions with robust standard errors clustered on physician participants. The dependent variable is top-1 accuracy, and the constant term represents primary care physicians' diagnostic accuracy. Column (1) includes the first 10 differential diagnoses of images by participants who passed the attention check and completed at least 10 differential diagnoses of images (2) includes the subset of participants from (1) who live in the United States (3) includes the subset of participants from (1) and also includes participants who provided fewer than 10 differential diagnoses of images, and (4) includes all responses of all physician participants who pass the attention check.The coefficients represent the change in the dependent variable for a one-unit change in the independent variable while holding everything else constant. The numbers in parentheses are standard errors indicating the variability of coefficient estimates. *, **, and *** indicates the p-value from the ordinary least squares regression is less than 0.05, 0.01, and 0.001.

	Dependent variable: PCP Top-3 Accuracy			
	(1)	(2)	(3)	(4)
Constant	0.20***	0.21***	0.21***	0.21***
	(0.01)	(0.01)	(0.01)	(0.01)
Fitzpatrick Skin Type 5 and 6	-0.04**	-0.05**	-0.04**	-0.05***
	(0.01)	(0.02)	(0.01)	(0.01)
Observations	3,150	2,052	3,352	4,999
Number of Primary Care Providers	350	228	434	441
Number of Images	363	363	363	363
R^2	0.00	0.00	0.00	0.00
Note:	*p<0.05; **p<0.01; ***p<0.001			

Supplementary Information Table 4: **Robustness Check for Regression of Patients' Fitzpatrick Skin Type on PCP Physicians' Top-3 Accuracy.**Robustness check for top-3 accuracy disparities across skin tone based on alternative inclusion criteria for primary care physicians. This table shows ordinary least squares regressions with robust standard errors clustered on physician participants. The dependent variable is top-3 accuracy, and the constant term represents primary care physicians' diagnostic accuracy. Column (1) includes the first 10 differential diagnoses of images by participants who passed the attention check and completed at least 10 differential diagnoses of images (2) includes the subset of participants from (1) who live in the United States (3) includes the subset of participants from (1) and also includes participants who provided fewer than 10 differential diagnoses of images, and (4) includes all responses of all physician participants who pass the attention check. The coefficients represent the change in the dependent variable for a one-unit change in the independent variable while holding everything else constant. The numbers in parentheses are standard errors indicating the variability of coefficient estimates. *, **, and *** indicates the p-value from the ordinary least squares regression is less than 0.05, 0.01, and 0.001.

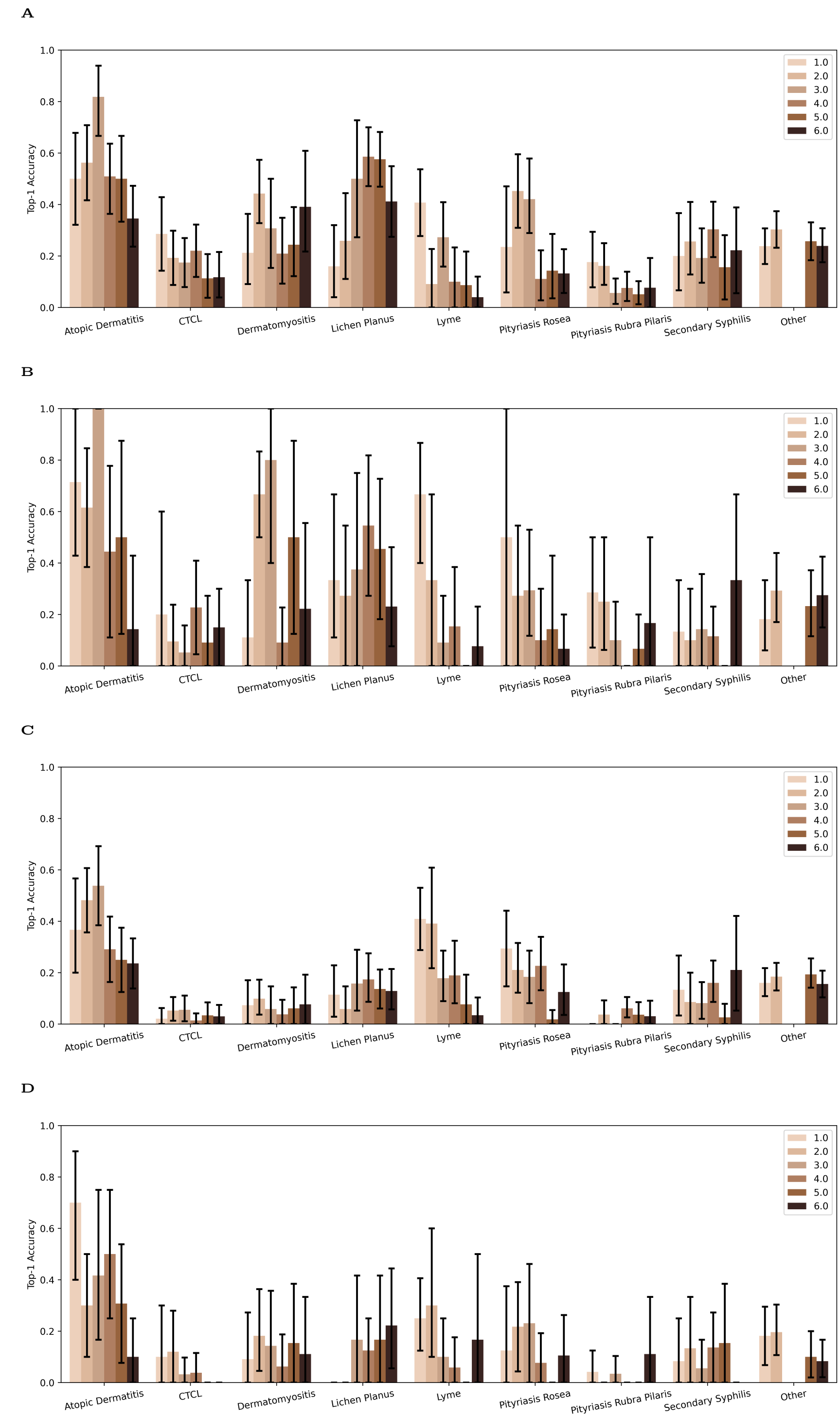

Supplementary Information Figure 6: Top-1 Diagnostic Accuracy by Physician Type, Skin Disease, and Fitzpatrick Skin Type. Top-1 accuracy of all physician types (A. BCDs, B. dermatology residents, C. PCPs, D. Other MDs/DOs) across skin diseases and Fitzpatrick skin types. The error bars represent the 95% confidence interval of the true mean.

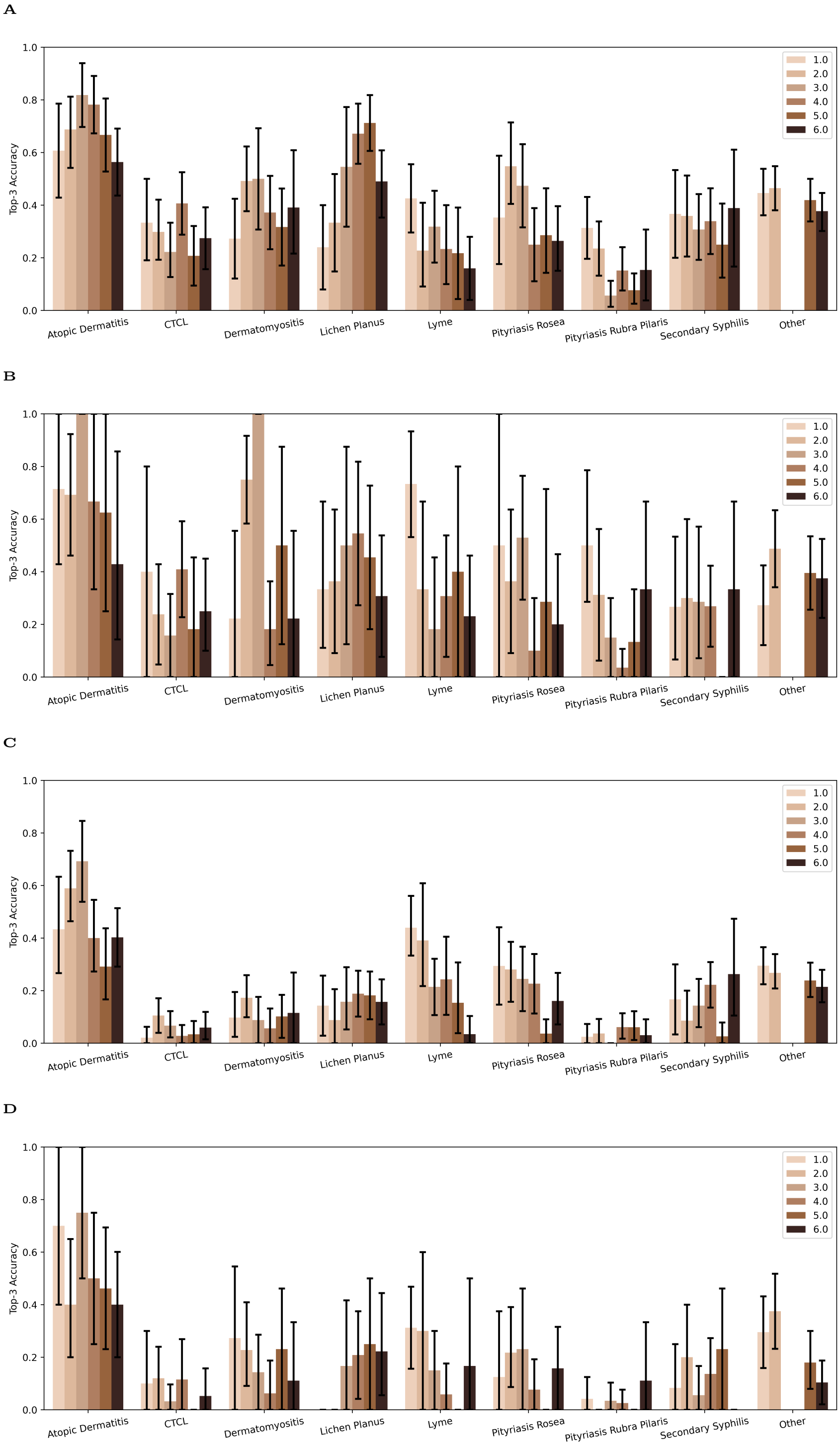

Supplementary Information Figure 7: Top-3 Diagnostic Accuracy by Physician Type, Skin Disease, and Fitzpatrick Skin Type. Top-3 accuracy of all physician types (A. BCDs, B. dermatology residents, C. PCPs, D. Other MDs/DOs) across skin diseases and Fitzpatrick skin types. The error bars represent the 95% confidence interval of the true mean.

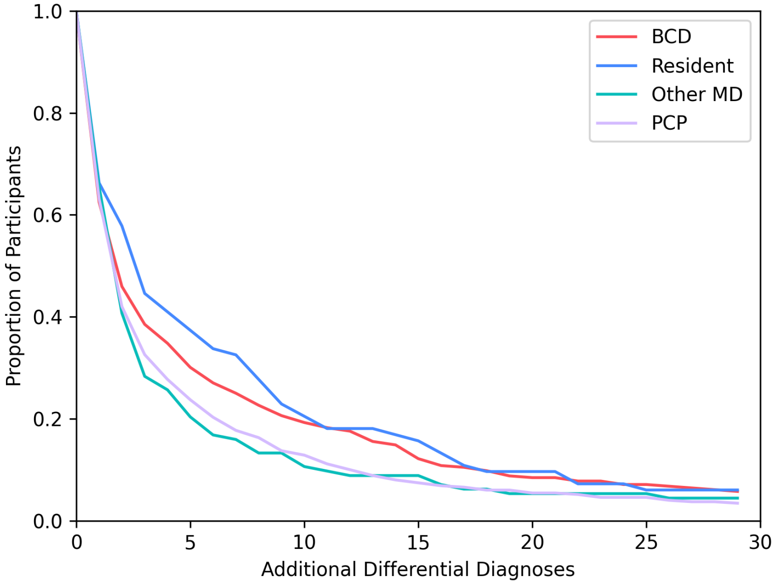

Supplementary Information Figure 8: **Continued Participation after Submitting 10 Sets of Differential Diagnoses.** 63% BCDs, 66% dermatology residents, 65% other physicians, and 63% PCPs continued participating in the experiment after finishing the 10 sets of differential diagnoses that were required for collecting compensation.
