# Supplementary material for: Deep learning-aided decision support for diagnosis of skin disease across skin tones
Source: Nat Med. 2024 Feb 5;30(2):573–83. doi: 10.1038/s41591-023-02728-3 (PMC10878981; doi:10.1038/s41591-023-02728-3)
Supplement: Supplementary file 2 — Reporting Summary [file 41591_2023_2728_MOESM2_ESM.pdf]

nature portfolio

Corresponding author(s): Matthew Groh

Last updated by author(s): Nov 3, 2023

Reporting Summary

Nature Portfolio wishes to improve the reproducibility of the work that we publish. This form provides structure for consistency and transparency in reporting. For further information on Nature Portfolio policies, see our Editorial Policies and the Editorial Policy Checklist.

Statistics

For all statistical analyses, confirm that the following items are present in the figure legend, table legend, main text, or Methods section.

n/a	Confirmed	
☐	☒	The exact sample size (*n*) for each experimental group/condition, given as a discrete number and unit of measurement
☐	☒	A statement on whether measurements were taken from distinct samples or whether the same sample was measured repeatedly
☐	☒	The statistical test(s) used AND whether they are one- or two-sided<br>*Only common tests should be described solely by name; describe more complex techniques in the Methods section.*
☐	☒	A description of all covariates tested
☐	☒	A description of any assumptions or corrections, such as tests of normality and adjustment for multiple comparisons
☐	☒	A full description of the statistical parameters including central tendency (e.g. means) or other basic estimates (e.g. regression coefficient) AND variation (e.g. standard deviation) or associated estimates of uncertainty (e.g. confidence intervals)
☐	☒	For null hypothesis testing, the test statistic (e.g. *F*, *t*, *r*) with confidence intervals, effect sizes, degrees of freedom and *P* value noted<br>*Give P values as exact values whenever suitable.*
☒	☐	For Bayesian analysis, information on the choice of priors and Markov chain Monte Carlo settings
☒	☐	For hierarchical and complex designs, identification of the appropriate level for tests and full reporting of outcomes
☐	☒	Estimates of effect sizes (e.g. Cohen's *d*, Pearson's *r*), indicating how they were calculated

Our web collection on statistics for biologists contains articles on many of the points above.

Software and code

Policy information about availability of computer code

Data collection	We hosted the store-and-forward digital experiment at https://diagnosing-diagnosis.media.mit.edu using a website built in Python using the Flask web framework. All data is collected based on how participants interact with the experiment.
Data analysis	The data analysis is available at https://researchbox.org/1802 was performed in Python 3.9.6 with the following libraries pandas 1.4.0, matplotlib 3.2.2, seaborn 0.11.1, numpy 1.18.5, scipy 1.5.0, statsmodels, stargazer 0.11.1, and sklearn 0.0.5.

For manuscripts utilizing custom algorithms or software that are central to the research but not yet described in published literature, software must be made available to editors and reviewers. We strongly encourage code deposition in a community repository (e.g. GitHub). See the Nature Portfolio guidelines for submitting code & software for further information.

Data

Policy information about availability of data

All manuscripts must include a data availability statement. This statement should provide the following information, where applicable:

- Accession codes, unique identifiers, or web links for publicly available datasets
- A description of any restrictions on data availability
- For clinical datasets or third party data, please ensure that the statement adheres to our policy

The experimental data to reproduce the results of this study are available on ResearchBox at https://researchbox.org/1802. The 364 images used in the experiment

are available at https://doi.org/10.5281/zenodo.10070478 to registered Zenodo users who agree to only use this dataset for scientific and medical purposes and delete this data from their device once their research is complete.

Research involving human participants, their data, or biological material

Policy information about studies with human participants or human data. See also policy information about sex, gender (identity/presentation), and sexual orientation and race, ethnicity and racism.

Reporting on sex and gender	We do not collect data on sex and gender because we did not expect differences in diagnostic accuracy of physicians based on sex or gender. Furthermore, we are unable to determine the sex or gender of patients in the majority of clinical images because the images are generally focused on a particular skin lesion.
Reporting on race, ethnicity, or other socially relevant groupings	We examine diagnostic accuracy across images of light and dark skin based on the Fitzpatrick Skin Type scale to identify potential accuracy disparities in physicians, the deep learning system (DLS), and physicians supported by the DLS. We also asked participants (who are all physicians) to self-report their level of experience and expertise with white and non-white patients to examine how experience and expertise with or without diverse patients influences diagnostic accuracy and diagnostic accuracy disparities.
Population characteristics	In this experiment, 40% of physicians have been practicing medicine for 20 years or more, 26% have been practicing for 10 to 20 years, 22% have been practicing for 2 to 10 years, 3% have been practicing for 0 to 2 years, and the rest are doing residencies, fellowships, or internships. In response to the question ``How would you describe the distribution of your patients' skin colors?'', 32% of participants responded about an equal portion of white and non-white patients, 43% responded mostly white patients, 2% responded all white patients, 15% responded mostly non-white, 7% responded all non-white patients, and 1% responded that the question is not applicable.
Recruitment	We recruited participants by word-of-mouth and direct emails by Sermo, a secure digital (online) platform designed for physician networking and anonymous survey research, to their verified physician network. Sermo sent emails to 7,900 BCDs and 10,000 PCPs and offered $10 for BCDs and $5 for PCPs to complete the survey. This is a large convenience sample of physicians, and participants may have self-selected into this study for a number of potential reasons: interest in contributing to scientific research, interest in collecting a cash reward for completing the experiment, interest in participating in research on dermatology diagnosis, and interest in participating in research on diverse skin tones. We have no evidence to suggest there is selection bias for these participants relative to board-certified dermatologists, dermatology residents, primary care physicians, and other physicians.
Ethics oversight	The Massachusetts Institute of Technology's Committee on the Use of Humans as Experimental Subjects determined this study to fall under Exempt Category 3 – Benign Behavioral Intervention. This study's exemption identification numbers are E-2875 and E-3675.

Note that full information on the approval of the study protocol must also be provided in the manuscript.

Field-specific reporting

Please select the one below that is the best fit for your research. If you are not sure, read the appropriate sections before making your selection.

☐ Life sciences ☒ Behavioural & social sciences ☐ Ecological, evolutionary & environmental sciences

For a reference copy of the document with all sections, see nature.com/documents/nr-reporting-summary-flat.pdf

Behavioural & social sciences study design

All studies must disclose on these points even when the disclosure is negative.

Study description	This is a quantitative experimental study.
Research sample	Our participants include board-certified dermatologists, dermatology residents, primary care physicians, and other physicians. This sample is a convenience sample and is not necessarily representative of all physicians. Nearly half of participants are based in the United States, and the other half come from countries across the world. We did not collect data on participants' sex or age, but we collected data on years of experience in medicine. In this experiment, 40% of physicians have been practicing medicine for 20 years or more, 26% have been practicing for 10 to 20 years, 22% have been practicing for 2 to 10 years, 3% have been practicing for 0 to 2 years, and the rest are doing residencies, fellowships, or internships. The main focus of this study is how accurately physicians diagnose skin conditions in store-and-forward teledermatology settings and how DLS assistance can (or cannot augment) their performance, and as such, we selected specialist and generalists physicians as the target research sample.
Sampling strategy	We used a convenience sample based on recruiting participants by word-of-mouth and direct emails by Sermo, a secure digital (online) platform designed for physician networking and anonymous survey research, to their verified physician network. Sermo sent emails to 7,900 BCDs and 10,000 PCPs and offered $10 for BCDs and $5 for PCPs to complete the survey. Our target sample size was 1,000 physicians and 10,000 observations based on Sermo's projections for how many participants would respond and a power calculation showing that a two independent sample study with a dichotomous endpoint and 95% power requires 4,188 observations to detect a 5 percentage point difference in groups assuming a baseline accuracy of 50%.

Data collection	We hosted the store-and-forward digital experiment at https://diagnosing-diagnosis.media.mit.edu and participants could complete the experiment on their personal computing device. Participants are blinded to the experimental conditions, and the researchers are not blinded to the experimental conditions or research hypothesis.
Timing	The experiment launched on March 16, 2022 and closed on December 30, 2022.
Data exclusions	The final dataset includes 28159 rows (14,261 observations of physicians' diagnoses and 14,258 observations of physicians' interaction with the deep learning system). We excluded 40 rows where participants responded "test" and 5 rows where a bug occurred such that the user id was malformed. In the results sections on diagnostic accuracy, we focus our analysis on the first ten differentials provided by participants who passed the attention check and provided at least 10 differentials. This includes 2,660 differentials from 296 BCDs, 747 differentials from 83 dermatology residents, 3,150 differentials from 350 PCPs, and 1,015 differentials from 113 other physicians. We show that our results are robust to other selection criteria such as only participants from the United States, participants who provided fewer than 10 differentials, and all participants who pass the attention check.
Non-participation	We define full participation as passing the attention check and providing differential diagnoses on at least 10 images. 76% of BCDs and PCPs, 73% of other physicians, and 72% of dermatology residents who started the experiment pass the attention check and provide differential diagnoses on at least 10 images.
Randomization	We conducted two randomized experiments where participants were assigned to control and treatment conditions. We randomly assigned participants to see suggestions from a control model (the 47% accurate model) or a synthetically enhanced treatment model (the 84% accurate model). We also randomly assigned the order in which the options appear for including or ignoring the suggestion in a participant's differential diagnosis. The treatment group saw ``Keep my differential'' on top and ``Update my top prediction with [condition]'' on the bottom whereas the control group saw the opposite where ``Update my top prediction with [condition]'' appeared on the top.

Reporting for specific materials, systems and methods

We require information from authors about some types of materials, experimental systems and methods used in many studies. Here, indicate whether each material, system or method listed is relevant to your study. If you are not sure if a list item applies to your research, read the appropriate section before selecting a response.

Materials & experimental systems

n/a	Involved in the study
☒	☐ Antibodies
☒	☐ Eukaryotic cell lines
☒	☐ Palaeontology and archaeology
☒	☐ Animals and other organisms
☒	☐ Clinical data
☒	☐ Dual use research of concern
☒	☐ Plants

Methods

n/a	Involved in the study
☒	☐ ChIP-seq
☒	☐ Flow cytometry
☒	☐ MRI-based neuroimaging
